# Antibacterial, antibiofilm and cytotoxic properties of *Aloe vera*-synthesized silver titanium nanoparticles

**DOI:** 10.1186/s13568-025-01965-8

**Published:** 2025-10-27

**Authors:** Mariam Z. Abouelwafa, Sarra E. Saleh, Mai S. A. Hussien, Ibrahim S. Yahia, Khaled M. Aboshanab

**Affiliations:** 1 Department of Microbiology and Immunology, Faculty of Pharmacy, Ain Shams University, Cairo, 11566 Egypt; 2 Department of Chemistry, Faculty of Education, Ain Shams University, Roxy, 11757 Cairo Egypt; 3 Green Research Laboratory (GRL), Faculty of Education, Ain Shams University, Roxy, 11757 Cairo Egypt; 4 Nanoscience Laboratory for Environmental and Bio-Medical Applications (NLEBA), Faculty of Education, Ain Shams University, Roxy, 11757 Cairo Egypt; 5 Laboratory of Nano-Smart Materials for Science and Technology (LNSMST), Department of Physics, Faculty of Science, King Khalid University, P.O. Box 9004, Abha, Saudi Arabia

**Keywords:** MDR, MRSA, *S*. Typhi, *K. pneumoniae*, NPs, Silver titanium dioxide NPs

## Abstract

The green synthesis of silver (Ag) and titanium dioxide (TiO_2_) nanoparticles (NPs) is of great interest due to their unique and controllable properties, as well as the eco-friendly and low-cost process involved. In this study, green-synthesized Ag, TiO_2_, and AgTiO_2_ NPs were prepared with four different ratios of TiO_2_: Ag (1:0.5, 1:1, 1:1.5, and 1:2) using *Aloe vera* (*A. vera*) as a reducing agent. NPs were characterized and examined against multidrug-resistant (MDR) bacteria, MRSA, *Klebsiella pneumoniae,* and *Salmonella* Typhi. The antibiofilm and cytotoxic effects of the respective NPs were also investigated. The NPs’ synthesis, morphology, crystalline structure, and coating were confirmed and characterized using X-ray diffraction, scanning electron microscopy, and Fourier transform infrared spectroscopy. Additional characterization, including transmission electron microscopy, high-resolution transmission electron microscopy, and selected area electron diffraction, was performed only on the best NPs showing the best biological effect. The produced NPs were found to be irregular spheres and polygonal in shape of 20–70 nm in size. AgTiO_2_ NPs showed superior antibacterial activity against MRSA, followed by *S*. Typhi, then *K. pneumoniae.* Their MIC values ranged from 250 to 15.62 µg/mL, and MBC values ranged from 1000 to 15.62 µg/mL, while the biofilm inhibition ranged from 79.5 to 96.6%. All NPs showed cytotoxic at different concentrations, showing more cytotoxicity against *Panc*-1 cells. The AgTiO_2_ (2:1) NPs were revealed to be the most effective and cytotoxic sample. In conclusion, AgTiO_2_ (2:1) NPs showed to be a promising candidate for clinical evaluation due to their tremendous in vitro biological activities.

**Supplementary Information:**

The online version contains supplementary material available at 10.1186/s13568-025-01965-8.

## Introduction

Antimicrobial resistance is considered by the World Health Organization (WHO) an urgent public threat among the top global public health threats (Mishra et al. 2022). In 2021, 4.71 million deaths were accompanied by antibacterial resistance. By 2050, AMR is estimated to cause 10 million deaths per year (Xu et al. 2025). The overuse and inappropriate use of antibacterial agents have resulted in the emergence of bacterial resistance (Nejatpour et al. 2025). Methicillin-resistant *Staphylococcus aureus* (MRSA) and *Klebsiella pneumoniae* are from the serious resistant bacteria (Azzam et al. 2023), and *Salmonella* Typhi is a life-threatening pathogen (Khan and Shamim 2022). In recent years, the use of NPs has emerged. They are novel, potent antibacterial agents showing efficacy against a diverse array of organisms due to exceptional physical and chemical properties (Hidayat et al. 2024; Uthiravel et al. 2024). There are many types of NPs: metallic (silver, gold, copper), inorganic, polymeric, and functionalized (Rao et al. 2025). Ag NPs of size < 100 nm and TiO_2_ NPs are widely used in the biomedical field for their promising antibacterial properties (Thakur et al. 2024; Mishra et al. 2022).

The antimicrobial properties of Ag NPs are based on the release of Ag ions, which interact with microbial enzymes and proteins, and combine electrostatically with the bacteria, changing their permeability, and generating highly reactive oxygen species (Rao et al. 2025; Zhou et al. 2024). While TiO_2_ NPs inhibit by producing ROS in microbial cells, causing lipid peroxidation of extracellular polymeric substances, and oxidation of internal enzymes, leading to apoptosis (Bereanu et al. 2024; Serov et al. 2024). Furthermore, by encouraging transport from the cell membrane to the target region, the use of NPs enables high bioavailability (Sorinolu et al. 2022). Masoudi et al. studied the antibacterial activity of TiO_2_ NPs and ZnO NPs alone, and associated with amoxicillin-clavulanic acid antibiotic, against MDR *A. baumannii* and *E. coli* strains. Pure forms of NPs showed high antibacterial activity, while associated with an antibiotic, it reduced the antibiotic’s effective dose by eightfold (Masoudi et al. 2024). Selim et al. studied the antioxidant, anticancer, and antibacterial effects of Ag-TiO_2_ NPs synthesized using leaf extract of *Pluchea indica* (Selim et al. 2025). The anticancer effect was tested against the Wi-38 normal cell line and the MCF-7 cancerous cell line. Moreover, the antibacterial activity was studied against *S. aureus* ATCC, *B. cereus* ATCC, *K. pneumonia*, *P. aeruginosa*, and *E. coli,* which showed promising results (Selim et al. 2025). Hojda et al. tested antibacterial and antibiofilm activity of titanium nitride alone and with silver nanoparticles against various ATCC strains, showing that TiN/Ag coating demonstrated superior activity over TiN NPs (Hojda et al. 2025). Rajkumari et al. synthesized TiO_2_ NPs using *A. vera* and were tested against *Pseudomonas aeruginosa,* causing zones of inhibition equal to 11and 14 mm, respectively, and showing MIC causing cell inhibition by 30.76 ± 3.96% (Rajkumari et al. 2019).

The plant-mediated NP biosynthesis has garnered considerable attention as a low-energy, low-cost, safe, and environmentally beneficial method (Thakur et al. 2024). Biomolecules from plants function as reducing, stabilizing, and capping agents in NP synthesis, with the added advantage of being non-toxic. Numerous medicinal benefits of miracle plant *A. vera* are widely recognized, including antimicrobial, anticancer, anti-ulcer, hepatoprotective, antioxidant, immunomodulatory, anti-inflammatory, and anti-diabetic effects (Rajkumari et al. 2019; García-García et al. 2025). Therefore, in this study, we aimed to evaluate the antibacterial, antibiofilm, and cytotoxic effects of *A. vera* green-synthesized AgTiO_2_ NPs against three serious and life-threatening MDR bacteria, including MRSA*, K. pneumoniae,* and *S.* Typhi serotype. Six samples were prepared using *A. vera* extract and characterized by various analytical techniques, four different AgTiO_2_ NPs due to changing silver content in each sample, pure TiO_2_ NP, and pure Ag NP. Using different silver concentrations makes studying antibacterial, antibiofilm, and cytotoxic effects clearer.

## Materials and methods

### Chemicals and materials

The *A. vera* plant was freshly collected from a local nursery (Cairo, Egypt), titanium isopropoxide (Sigma-Aldrich, Saint Louis, MO, USA), and silver nitrate AgNO_3_ (Sigma-Aldrich, Saint Louis, MO, USA).

### Preparation of *Aloe vera* extract

The preparation of *A. vera* extract was carried out as described earlier (Rajkumari et al. 2019; Mahardika et al. 2021), with some modifications. The *A. vera* plant was freshly collected from a local nursery (Cairo, Egypt), washed with water to remove dust, then with deionized water, and cut into small pieces. Different amounts were weighed: 1, 25, 50, and 100 g, then ground. About 100 mL of deionized water was added to each weight and stirred for three hours at 60 °C, then filtered using Whatman paper and kept in a refrigerator at 4 °C.

### Synthesis of TiO_2_ NPs

With some modifications, TiO_2_ NPs were prepared as reported in Rajkumari et al. (2019). About 30 mL (1 M) titanium isopropoxide (Sigma-Aldrich, Saint Louis, MO, USA) was added to 100 mL deionized water under constant stirring, then the prepared *A. vera* extract (1, 25, 50, and 100 mg/100 mL) was added dropwise, and pH was adjusted to 7, then was left to stir for three hours. Afterwards, sonication (20 kHz, 220 V) was done for 30 min. The produced NPs were washed, dried, and finally calcined at 550 °C for 4 h. Four samples were produced according to the concentration of *A. vera* extract: TiO_2_ (1), TiO_2_ (25), TiO_2_ (50), TiO_2_ (100). The four samples of TiO_2_ NPs were tested against *E. coli* ATCC 25922 and *S. aureus* ATCC 25923 to measure their antibacterial activity in order to combine the best sample with Ag NPs.

### Synthesis of Ag NPs

All steps were done in the dark. About 4.25 mg of AgNO_3_ (Sigma-Aldrich, Saint Louis, MO, USA) was added to 100 mL of deionized water (0.25 M AgNO_3_), stirred for 20 min to help dissolve AgNO_3_, then the *A. vera* extract (100 g/100 mL) was added dropwise, and the pH was adjusted to 7. The mixture was stirred for 24 h at 60 °C, then added to the microwave for 5 min (500 watts). The produced NPs were washed, dried, and then calcined at 550 °C for 4 h. Ag NPs synthesis took place as previously described by Albulym et al. (2021).

### Synthesis of AgTiO₂ NPs

The prepared TiO_2_ NPs (that showed maximum antibacterial activity against *E. coli* ATCC 25922 and *S. aureus* ATCC 25923) were added to different concentrations of silver nitrate (AgNO_3_) to produce AgTiO_2_ NPs in a ratio of TiO_2_: Ag (1:0.5, 1:1, 1:1.5, and 1:2). About 0.8, 1.6, 2.4, 3.2 g of silver nitrate was added to 100 mL of deionized water, stirred for 20 min to help dissolution of AgNO_3_. 1 g of TiO_2_ (100) NPs was added to each AgNO_3_ concentration, then the *A. vera* extract (100 g/100 mL) was added dropwise, and the pH was adjusted to 7. The mixture was left to stir for 24 h at 60 °C, then added to the microwave for 5 min (500 watts). The resulting NPs were washed, dried, and then calcined at 550 °C for 4 h. Four samples were produced AgTiO₂ (0.5:1), AgTiO₂ (1:1), AgTiO₂ (1.5:1), and AgTiO₂ (2:1) NPs.

### Characterization of NPs

Fourier Transform infrared (FTIR) spectrum was recorded by using the Perkin Elmer spectrophotometer (400–4500 cm^−1^). In addition, scanning electron microscopy (SEM) was performed using ThermoFisher Scientific (Waltham, Massachusetts, USA) Quattro S Field Emission Gun with Low Vacuum Secondary Electron Detector (20–200 Pa) at 15 kV acceleration voltage to inspect the NPs surface morphology. X-ray diffraction (XRD) was used to study the crystal structure and identify the phase of the NPs produced. The XRD examined the structure using a Panalytical Empyrean 3 X-ray diffractometer equipped with CuKα monochromatic radiation operated at (40 kV and 40 mA), measurement was recorded between 10° to 80° (2θ). The XRD patterns were collected in a Bragg–Brentano geometry. Further characterization was performed for the best-coated NPs. Transmission electron microscopy pictures were captured by Jeol (JEM-2100 PLUS, Akishima, Tokyo, Japan) at an accelerating voltage of 200 kV to estimate the NPs average size, Selected area electron diffraction (SAED) pattern confirmed the samples’ crystallinity, Energy Dispersive X-ray spectroscopy (EDS) and Mapping for elemental composition study.

### Bacterial isolates

The multidrug-resistant (MDR) MRSA coded CCASU-11*, K. pneumoniae* coded CCASU-24*,* and *S.* Typhi serotype coded CCASU-31 (collected from the Culture collection Ain Shams University (CCASU) (https://ccinfo.wdcm.org/collection/by_id/1186), were used for testing the antibacterial activities of Ag NPs, TiO₂ NPs, and AgTiO₂ NPs. All strains were subcultured in LB broth or agar and kept at 37 °C overnight. Stock cultures were maintained using LB broth with glycerol 80% and kept at − 20 °C for subsequent use. *E. coli* ATCC 25922 and *S. aureus* ATCC 25923 were used for testing the antibacterial activity of TiO₂ NPs.

### Evaluation of the antibacterial activities of the synthesized NPs

The agar well diffusion method, previously reported by Arshad et al. (2022), was employed to evaluate the antibacterial activity of the synthesized NPs against MDR isolates: MRSA CCASU-11, *K. pneumoniae* CCASU-24, and *S.* Typhi serotype CCASU-31. Sterilized/autoclaved Muller Hinton Agar (MHA) (Difco Laboratories, Detroit, USA) was poured in Petri plates and left to solidify under aseptic conditions. Eight wells were punched using sterile 6 mm corkborer and each well was filled with 100 µL of the tested NPs Ag, TiO₂ (100), AgTiO₂ (0.5:1), AgTiO₂ (1:1), AgTiO₂ (1.5:1), AgTiO₂ (2:1) (NPs were prepared in distilled water, 10 mg/mL), and ciprofloxacin (10 µg/mL) as positive control and distilled water as a negative control. All the plates were then incubated at 37 °C for 24 h. After incubation, the average inhibition zones (IZ in mm) were recorded. The same procedure was performed for the four TiO₂ NPs treated with different concentrations of *A. vera* extract (1, 25, 50, and 100 g/100 mL) against *S. aureus* ATCC 25923 and *E. coli* ATCC 25922. Gentamicin was used as a positive control, and DMSO as a negative control. This was performed to select the optimum TiO₂ NP sample to be coated with Ag NPs.

### Estimating the minimum inhibitory concentration (MIC) and the minimum bactericidal concentration (MBC)

The broth microdilution technique was performed for determining the MIC. Two-fold serial dilutions of Ag, TiO₂ (100), AgTiO₂ (0.5:1), AgTiO₂ (1:1), AgTiO₂ (1.5:1), and AgTiO₂ (2:1) NPs (31.25–1000 µg /mL) were prepared. The microbial suspension of MDR strains, MRSA, *K. pneumoniae*, and *S.* Typhi serotype was prepared in a concentration 1.5 × 10^8^ CFU/mL (0.5 McFarland standard). A 96-well plate containing nutrient broth was inoculated with about 10 μL of microbial suspension and 100 μL of NPs dilutions. The well containing nutrient broth only was used as a negative control, while the well with no NPs was used as a positive control. The 96-well plate was incubated at 37 °C for 24 h (Rajkumari et al. 2017; Parvekar et al. 2020). The MBC is determined depending on the previous MIC; 10 μL from the wells with no turbidity were seeded on nutrient agar plates and incubated for a further 24 h at 37 °C. The MBC endpoint is the lowest concentration of NPs at which 99.9% of the bacterial population is killed (Parvekar et al. 2020).

### Evaluation of the biofilm inhibition activities of the synthesized NPs

Microtiter Plate Assay was used for biofilm inhibition assessment of Ag, TiO₂ (100), AgTiO₂ (0.5:1), AgTiO₂ (1:1), AgTiO₂ (1.5:1), AgTiO₂ (2:1) NPs against MDR strains; MRSA, *K. pneumoniae*, and *S.* Typhi serotype. About 300 μL of inoculated fresh tryptic soy broth with test bacteria (1.5 X 10^8^ CFU/mL, 0.5 McFarland standard) was aliquoted into wells of a 96-well microtiter plate in the presence of different concentrations of NPs (75%, 50%, and 25% of MBC). The well containing tryptic soy broth only acted as a negative control, while the well without NPs acted as positive control. The plate was incubated at 37 °C for 24 h. The planktonic cells were gently rinsed with phosphate saline buffer (PBS, 0.1%), the microtiter plate was then air-dried and treated with crystal violet (0.1%). The excess dye was decanted, and the Microtiter Plate (MTP) was further rinsed twice with sterile distilled water to remove the unbound dye. The crystal violet attached to the biofilm was released using 1 mL ethanol (95%), and the absorbance was recorded at 600 nm. The percentage of biofilm inhibition was determined using the following equation (Eq. 1) (Antunes et al. 2010).

1 $$ \begin{aligned} & {\text{Percentage}}\;{\text{of}}\;{\text{biofilm}}\;{\text{inhibition}}\;\left( \% \right) = \left( {{\text{OD}}\;\left( {{\text{control}}} \right)} \right. \\ & \left. {\quad \quad \quad \quad \quad - {\text{OD}}\;\left( {{\text{treated}}} \right){\text{/OD}}\;\left( {{\text{control}}} \right)} \right) \times 100 \\ \end{aligned} $$

### Cytotoxic effect of NPs

The MTT Assay was performed as described previously (van de Loosdrecht et al. 1994; Afşar and Oltulu 2023) to assess Ag, TiO₂ (100), AgTiO₂ (0.5:1), AgTiO₂ (1:1), AgTiO₂ (1.5:1), and AgTiO₂ (2:1) NPs cytotoxic effect against *Vero* cell line (African Green Monkey) and *Panc-1* cell line (Human pancreatic ductal adenocarcinoma) (VACSERA, Giza, Egypt). Two-fold serial dilutions of NPs were prepared in distilled water (31.25–1000 μg/mL). About 100 μL (10.^5^ cells / mL) of cells were seeded in a 96-well plate with growth medium to form a monolayer. After incubation at 37 °C for 24 h, the medium was replaced with fresh medium containing 100 μL of NPs dilutions, leaving wells untreated with NPs as a negative control. The cells were further incubated at 37 °C for 24 h, then 20 μL 3-[4,5-dimethylthiazol-2-yl]-2,5-diphenyl tetrazolium bromide (MTT) (Sigma, St. Louis, MO, USA) solution was added to each well. Placed on a shaking table, 150 rpm for 5 min, to ensure thorough incorporation of MTT solution into the media. Incubate (37 °C, 5% CO_2_) for 4 h to allow the MTT to be metabolized. Dump off the media dry plate on paper towels to remove residue if necessary. Re-suspend formazan (MTT metabolic product) in 200 μL DMSO. Place on a shaking table, 150 rpm for 5 min, to thoroughly mix the formazan into the solvent. Read optical density at 540 nm. The percentage of cell viability was calculated using the following equation (Eq. 2)

2 $$ \begin{aligned} & {\text{Cell}}\;{\text{viability}}\;{\text{percentage}}\;\left( \% \right) = \left( {{\text{OD}}\;\left( {{\text{control}}} \right)} \right. \\ & \left. {\quad \quad \quad - {\text{OD}}\left( {{\text{treated}}} \right){\text{/OD}}\;\left( {{\text{control}}} \right)} \right) \times 100 \\ \end{aligned} $$

## Results

### Characterization of nanoparticles

#### X-ray diffraction (XRD) analysis

**Fig. 1:**
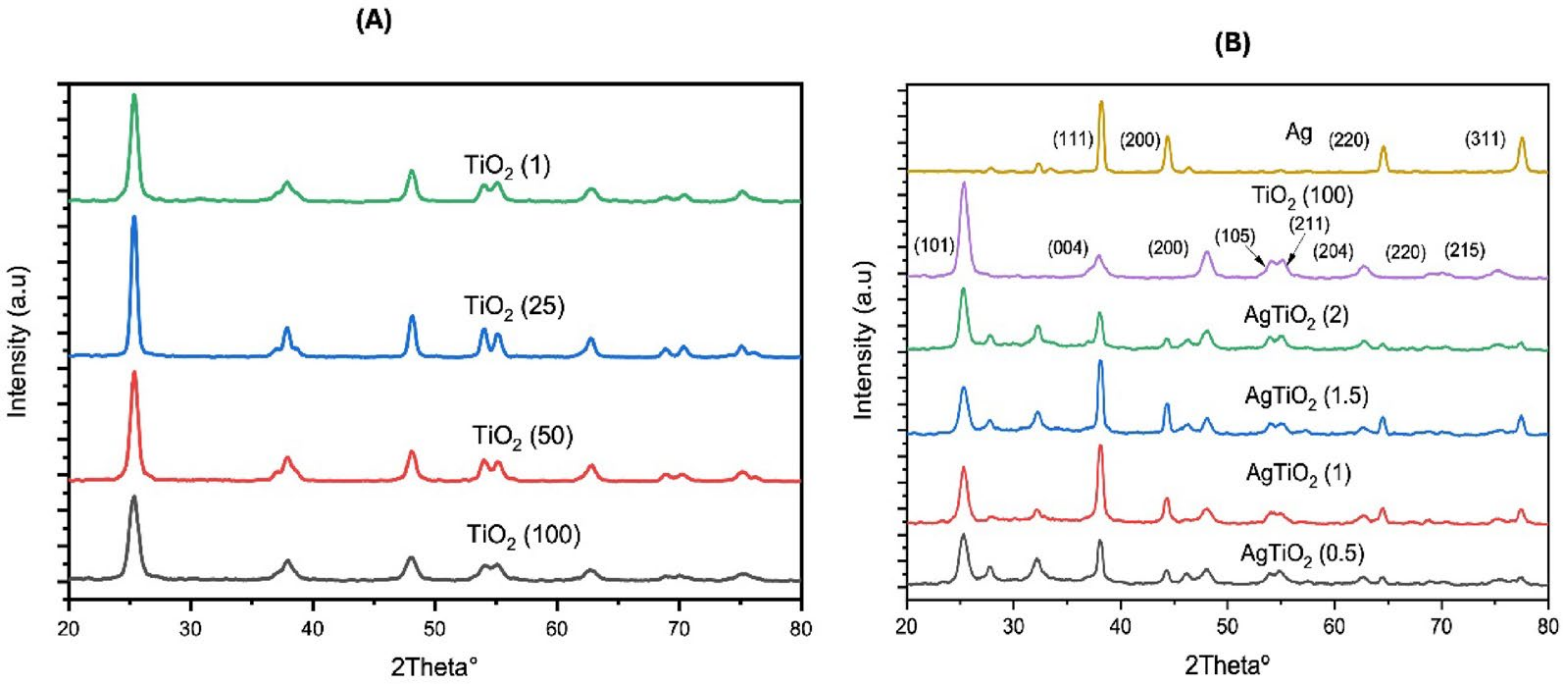
X-ray diffraction (XRD) analysis graph of: **A** TiO_2_ (1), TiO_2_ (25), TiO_2_ (50), TiO_2_ (100) NPs; **B** XRD graph of Ag, TiO₂ (100), AgTiO₂ (0.5:1), AgTiO₂ (1:1), AgTiO₂ (1.5:1), AgTiO₂ (2:1) NPs

The XRD explores crystallinity, crystal size, and NPs formation. Figure 1a shows the XRD patterns of TiO_2_ (1), TiO_2_ (25), TiO_2_ (50), TiO_2_ (100) NPs, while Ag, TiO₂ (100), AgTiO₂ (0.5:1), AgTiO₂ (1:1), AgTiO₂ (1.5:1), AgTiO₂ (2:1) NPs are shown in Fig. 1b. The x-axis represents two theta and the y-axis the intensity. Ag NPs showed diffraction peaks of planes (111), (200), (220), (311) at 2 theta 38.1°, 44.3°, 64.5°, 77.4° showing cubic structure, which had the same peak positions as silver JCPD Card (No. 01–087-0718), In a similar report Kumar et al. (Kumar et al. 2022) synthesized Ag NPs showing the same 2 theta XRD pattern. The peaks 27.9°, 32.3°, 46.3° found in Ag NPs XRD graph, as mentioned in many other reports, may be described as peaks due to the organic compounds found in the used plant extract (Roopan et al. 2013; Ibrahim 2015; Rautela et al. 2019). All TiO_2_ NPs XRD graphs showed diffraction peaks of planes (101), (004), (200), (105), (211),(204), (220), (215) at 2 theta 25.2°, 37.9°, 48°, 53.5°, 55.1°, 62.7°, 70.2°, 75.4° correlating with peaks of anatase TiO_2_ JCPD Card (No. 00-021-1272) showing tetragonal structure as documented in Kang et al. study (2022). After coating, all peaks of TiO_2_ and Ag NPs appeared. The peak at 2 theta 25.2° of TiO_2_ NPs decreased in intensity, while some other peaks as 27.9°, 32.3°, 46.3° appeared clearer than in the pure form of Ag NPs. The crystallite domain size, dislocation density, and lattice strain results of the prepared NPs are displayed in Table S1. The crystal size of the NPs was determined using the Debye–Scherrer equation, as shown below (Eq. 3):

3 $$ D = 0.9\lambda {/}\beta \cos \theta $$

In this equation, *D* represents the crystal size, 0.9 is the Debye–Scherrer constant, *β* is the full width at half maximum (FWHM), *θ* is the Bragg’s angle, and *λ* denotes the X-ray wavelength (Oleiwi et al. 2024).

Dislocation density (δ) was obtained through:

4 $$\updelta =\frac{1}{{\text{D}}^{2}}$$

While lattice strain (ε) was determined via:

5 $$ \upvarepsilon = \frac{\upbeta \cos \uptheta }{4} $$

#### Fourier transform infrared (FTIR)

**Fig. 2:**
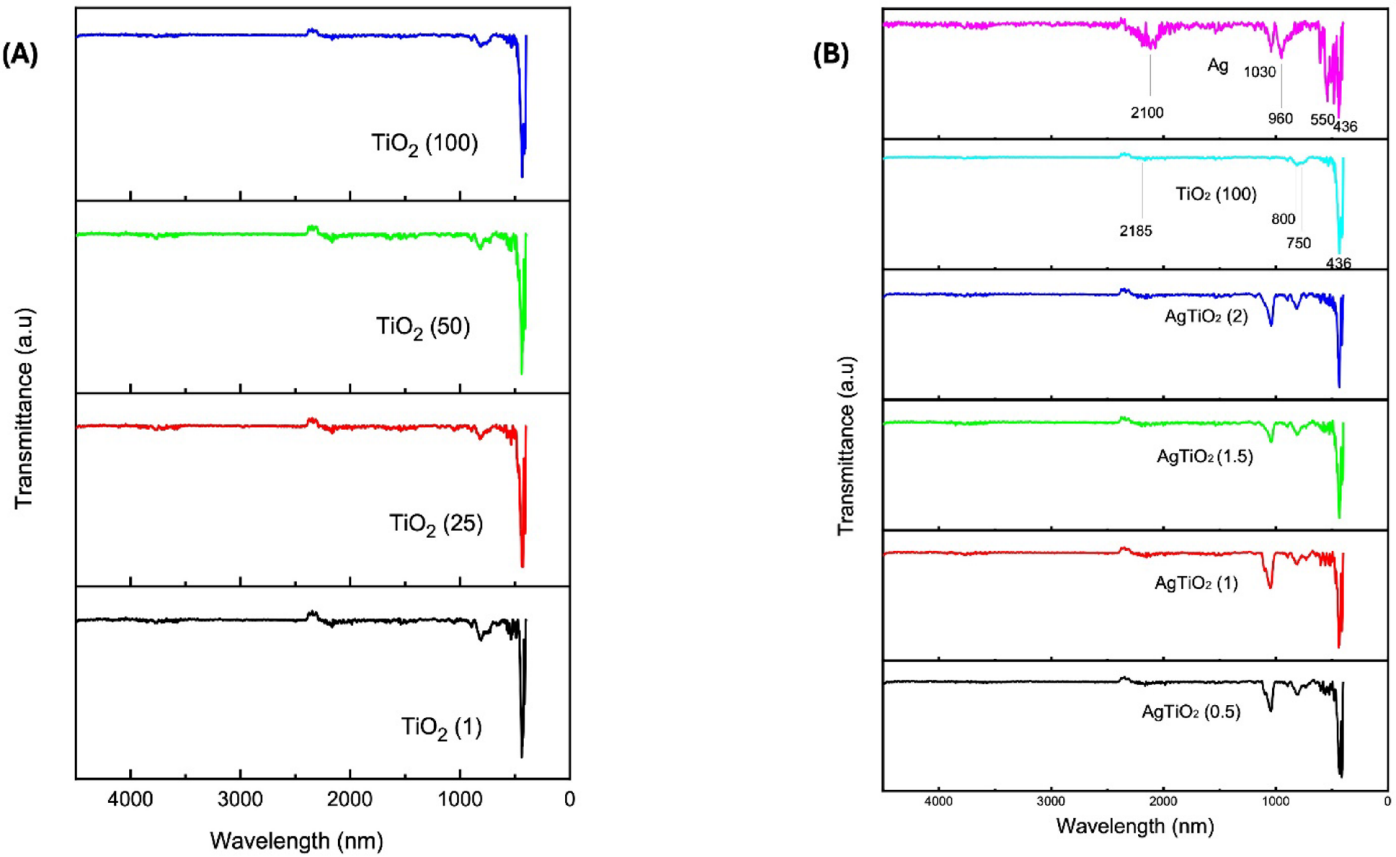
Fourier Transform infrared (FTIR) spectra of: **A** TiO_2_ (1), TiO_2_ (25), TiO_2_ (50), TiO_2_ (100) nanoparticles (NPs); **B** Ag, TiO₂ (100), AgTiO₂ (0.5:1), AgTiO₂ (1:1), AgTiO₂ (1.5:1), AgTiO₂ (2:1) NPs

The FTIR spectrum facilitates the identification of functional groups present in the synthesized NPs (Rajkumari et al. 2019). Figure 2A presents FTIR spectra of TiO_2_ (1), TiO_2_ (25), TiO_2_ (50), and TiO_2_ (100) NPs, and Fig. 2B presents FTIR spectra of Ag, TiO_2_ (100), AgTiO_2_ (0.5:1), AgTiO_2_ (1:1), AgTiO_2_ (1.5:1), and AgTiO_2_ (2:1) NPs. Results showed that no difference in peak intensity or position was observed between all TiO_2_ NPs. The sharp peak that appeared at 436 cm^−1^ indicates the Ti–O-Ti (metal oxide) lattice stretching vibration of anatase TiO_2_ as previously reported (Kelany et al. 2023; Wellia et al. 2024), while Elqady et al. (2025) reported that, Ti–O-Ti lattice stretching vibrations appeared at 656 cm^−1^. The bands at 750 cm^−1^ and 800 cm^−1^ are also attributed to the Ti–O-Ti (metal oxide) lattice stretching vibration. According to Kunnamareddy et al. Peaks in range of 400-900v cm^−1^ are attributed to Ti–O-Ti lattice stretching vibrations (Kunnamareddy et al. 2018). The peak at 2185 cm^−1^ indicated the presence of C = C stretching vibration of the aromatic ring, which likely represents the organic compounds in *A. vera* (Wellia et al. 2024). As for Ag NPs, the peaks appearing at 436 cm^−1^ and 550 cm^−1^ are attributed to Ag–O stretching and bending vibrations (Rathi et al. 2023), while the peak at 960 cm^−1^ is attributed to = C-H bending vibrations (Kelany et al. 2023), the peak at 1030 cm^−1^ represents the C–N stretching vibrations of primary or secondary amines (Mdluli et al. 2025), and the peak at 2100 cm^−1^ indicates the C = C stretching vibration of the aromatic ring (Wellia et al. 2024). Peaks 960 cm^−1^ and 1030 cm^−1^, and 2100 cm^−1^ in the Ag NP spectrum appear more intense than those in the TiO_2_ (100) NP spectrum. After coating, all peaks appeared clear with no difference except that the peak at 2100 appeared of low intensity (Zhang et al. 2018; Uthiravel et al. 2024).

#### Scanning electron microscope (SEM)

**Fig. 3:**
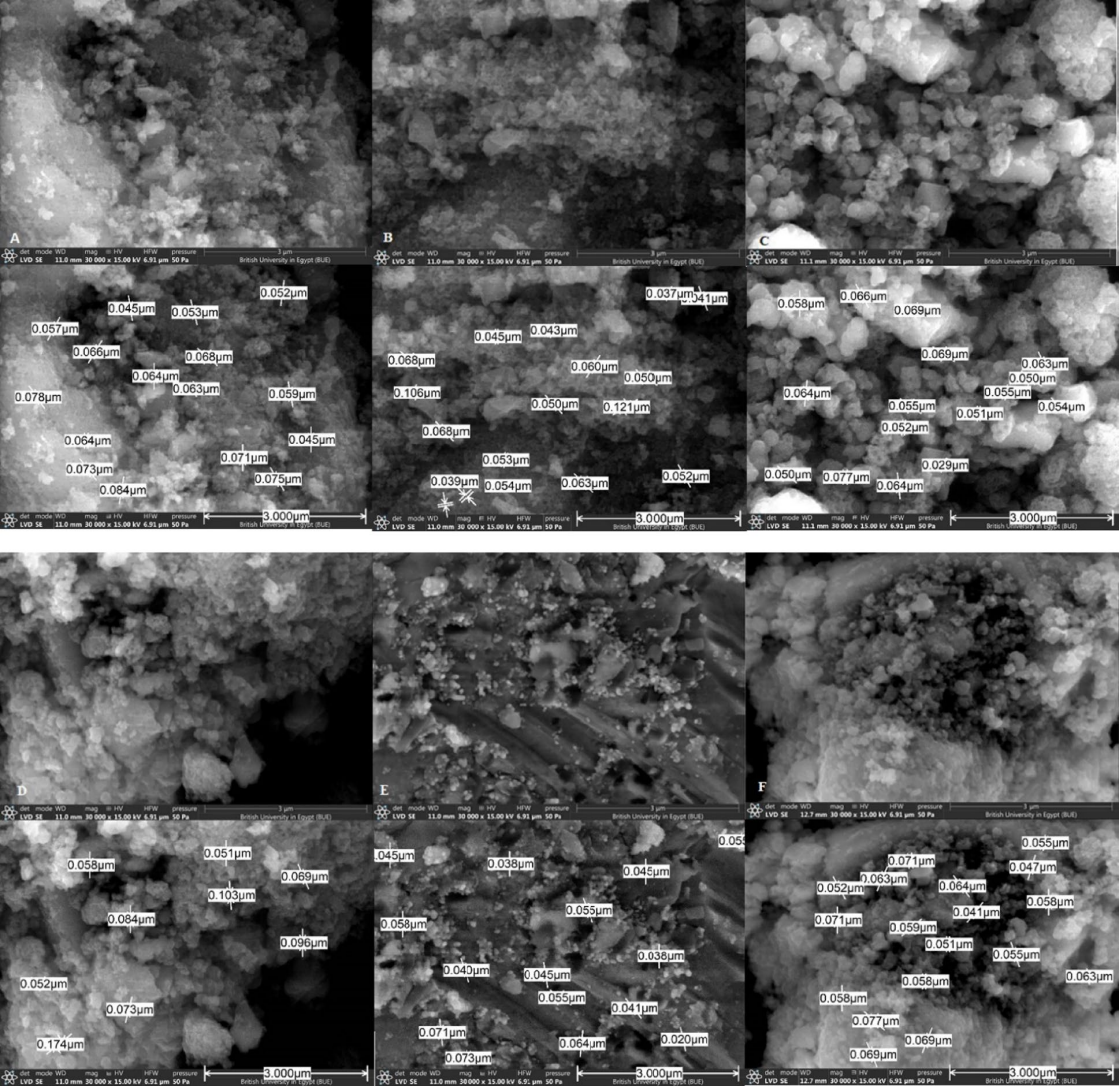

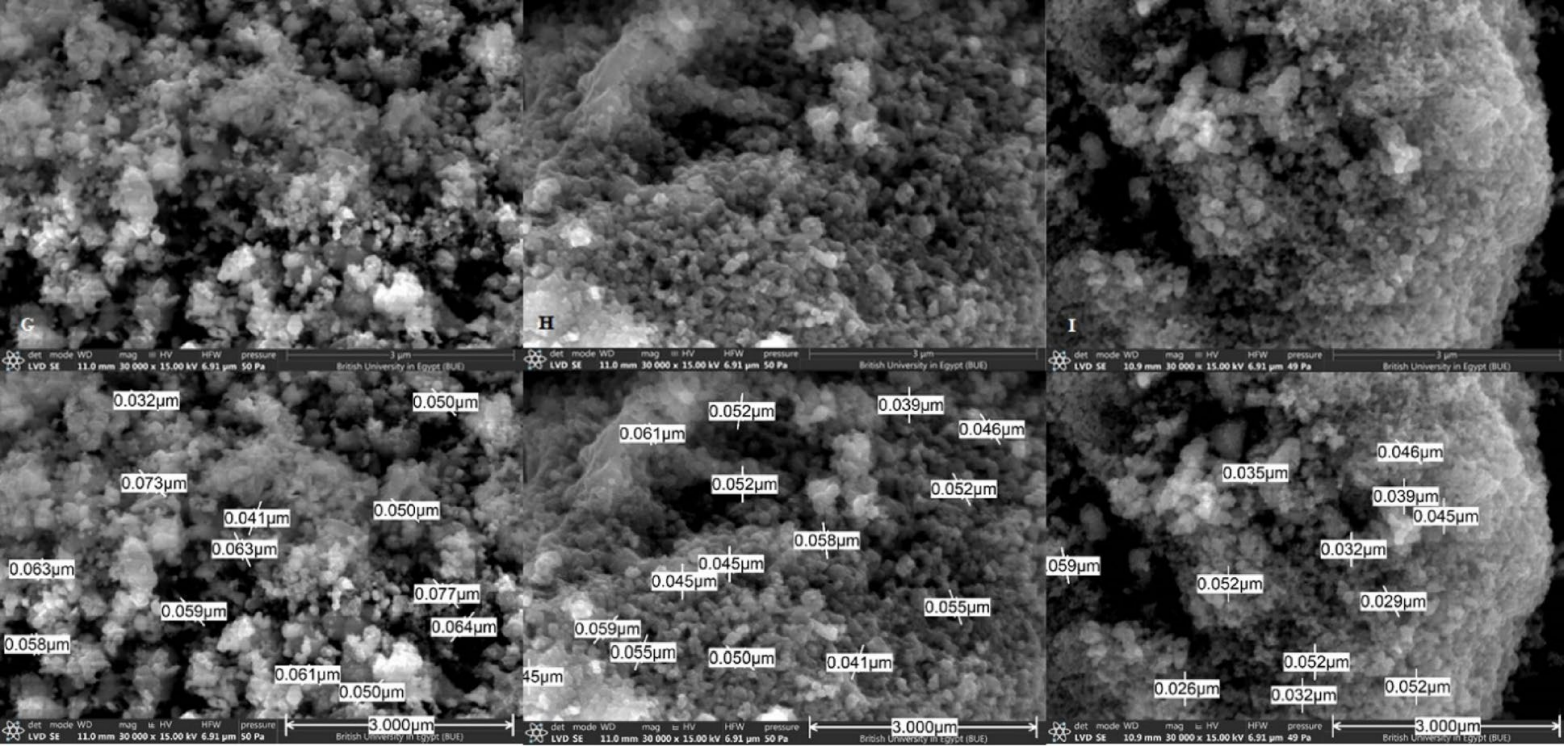
Scanning electron microscope (SEM) analysis of different green synthesized nanoparticles (NPs) and their grain size measurement **A** TiO_2_ (1) **B** TiO_2_ (25) **C** TiO_2_ (50) **D** TiO_2_ (100) **E** Ag **F** AgTiO_2_ (0.5:1) **G** AgTiO_2_ (1:1) **H** AgTiO_2_ (1.5:1) **I** AgTiO_2_ (2:1)

Scanning electron microscope (SEM) analysis studies surface topography, since the size and shape of NPs are important factors that affect the antibacterial activity. Analysis revealed polygonal particles (Fig. 3A–I). The NPs average size was calculated from SEM images by employing Smileview software, showing Ag NPs size of approximately 49 nm (Fig. 3E), similar to Kumar et al., who prepared green Ag NPs ranging from 20–80 nm (Kumar et al. 2022). TiO_2_ NPs ranged approximately from 50–70 nm (Fig. 3A–D), Ramya et al. reported TiO_2_ of 40–50 nm size (Ramya et al. 2024). AgTiO_2_ NPs ranged approximately from 20–70 nm (Fig. 3F–I), similar to Rathi et al. findings (Rathi et al. 2023).

#### Transmission electron microscopy (TEM), High resolution Transmission electron microscopy (HRTEM) and selected area electron diffraction (SAED) pattern investigation

**Fig. 4:**
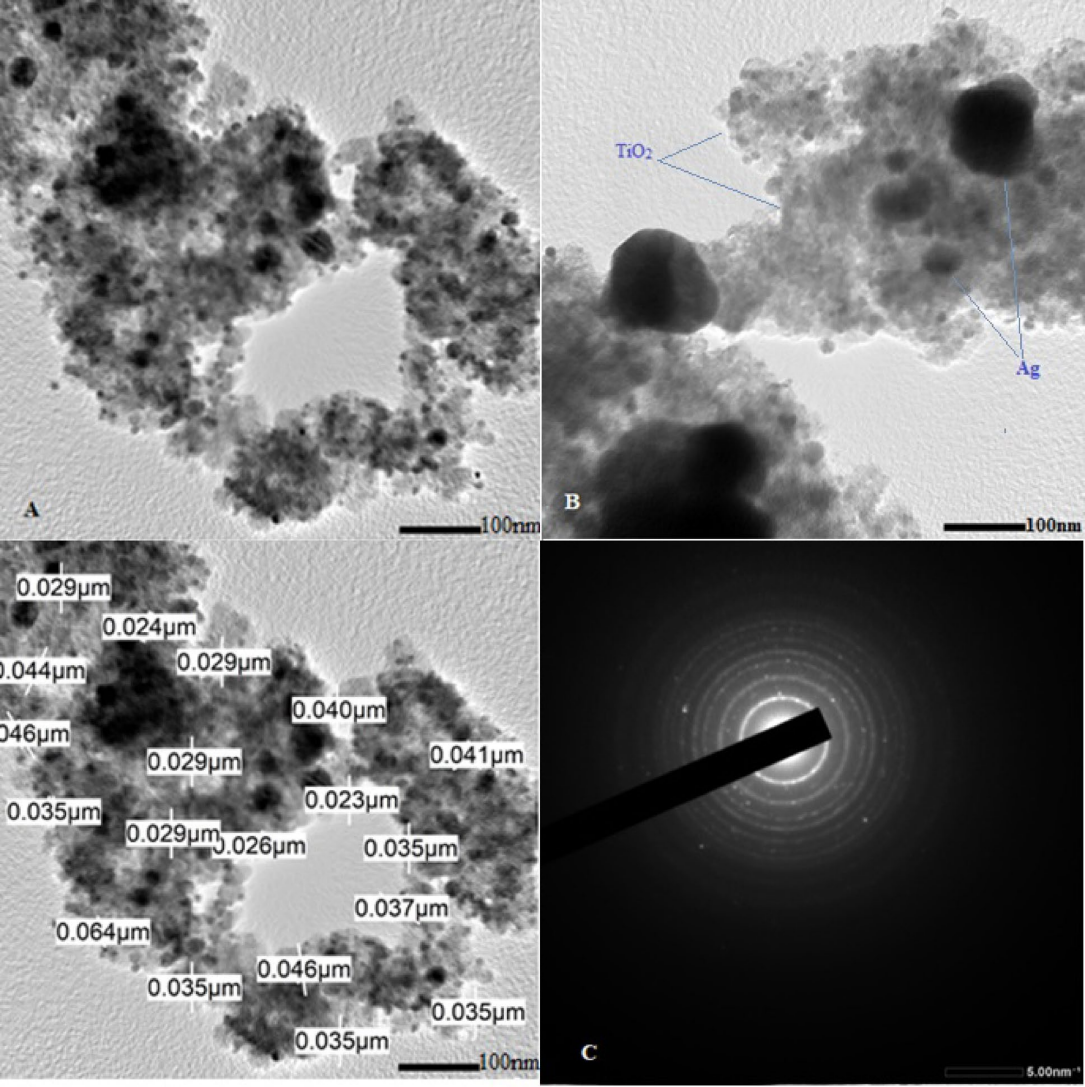
Transmission electron microscopic (TEM) analysis of AgTiO_2_ (2:1) nanoparticles (NPs) and its grain size measurement (**A**), high-resolution transmission electron microscopic (HRTEM) analysis of AgTiO_2_ (2:1) NPs (**B**); and selected area electron diffraction (SAED) pattern of AgTiO_2_ (2:1) NP (C)

Transmission electron microscopy/ High resolution Transmission electron microscopy estimated the shape and average size of synthesized AgTiO_2_ (2:1) NPs as depicted in (Fig. 4A, B). The images exhibited good agreement with previous SEM and XRD analysis (Nagaraj et al. 2023), showing polygonal shapes and irregular spheres of 35 nm average size. TEM images confirm successful coating of Ag NPs on TiO_2_ (100) NPs. The selected area electron diffraction (SAED) pattern confirmed the samples crystallinity and assured lattice orientation of Ag and TiO_2_ NPs. The definite circular fringes with dots confirmed the polycrystallinity of NPs (Fig. 4C).

#### Energy-dispersive X-ray spectroscopy (EDS)

**Fig. 5:**
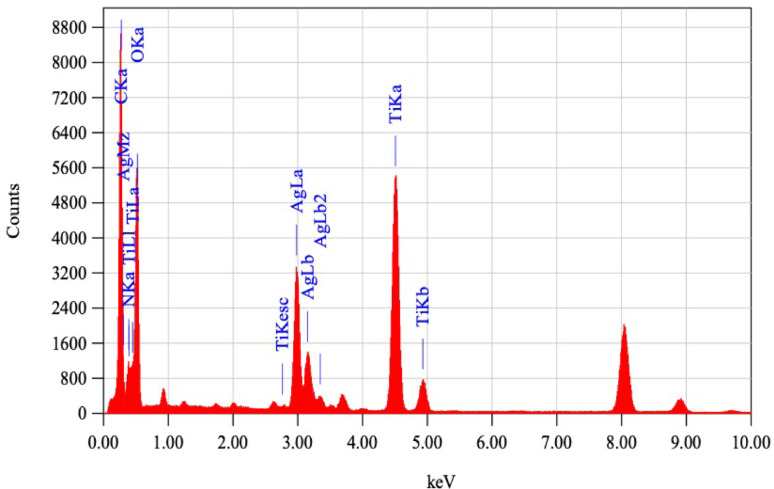
Energy-dispersion x-ray spectroscopy (EDS) of AgTiO_2_ (2:1) nanoparticles (NPs)

**Fig. 6:**
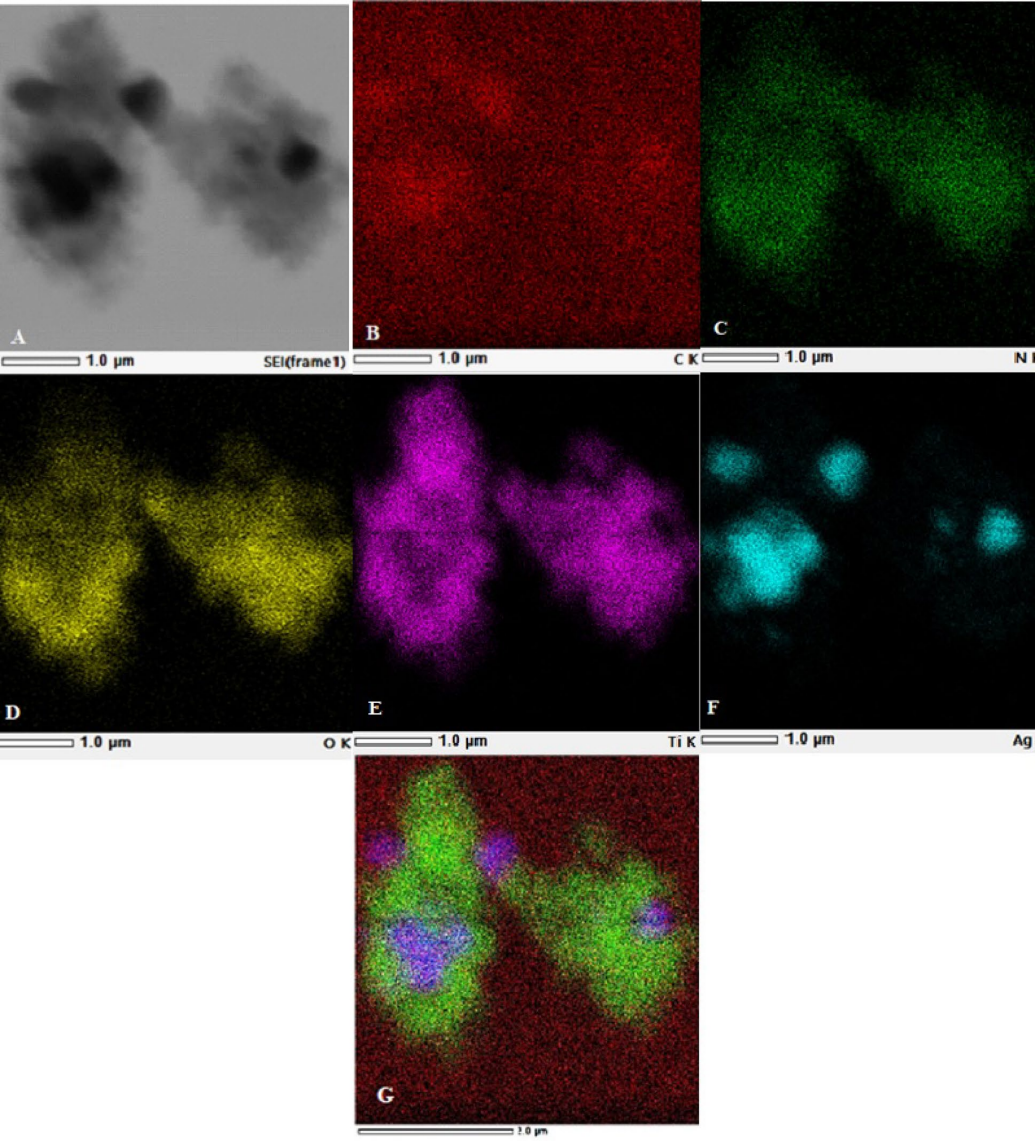
TEM elemental mapping: **A** High resolution TEM of AgTiO_2_ (2:1) nanoparticles (NPs); **B** Red colour represents carbon; **C** green nitrogen; **D** yellow oxygen; **E** purple titanium; **F** blue for silver; and **G** overview of all elements

Energy-dispersive X-ray spectroscopy (EDS) analysis studies elemental composition. It confirmed the presence of 57.31% silver, 29.47% titanium, 10.01% oxygen, 3.02% carbon, and 0.01% nitrogen at the same binding energies as (Hariharan et al. 2020). Carbon is from carbon coated grade used in capturing sample by Jeol (JEM-2100 PLUS, Akishima, Tokyo, Japan), and part of the carbon is from *A. vera.* While nitrogen is from *A. vera* used (Hossain et al. 2020) as shown in (Fig. 5). The elemental map distribution of AgTiO_2_ (2:1) NPs is shown in (Fig. 6), indicating the presence of Ag NPs as black spots on TiO_2_ NPs (Zhang et al. 2018). So, successful coating took place with approximately the right percentage (2:1) TiO_2_: Ag NPs.

### Evaluation of the antibacterial activities of the synthesized NPs

The TiO_2_ (1), TiO_2_ (25), TiO_2_ (50), and TiO_2_ (100) NPs were tested against *E. coli* ATCC 25922 and *S. aureus* ATCC 25923 strains to find the NP with the best antibacterial activity to be used in coating with Ag NPs. The TiO_2_ (100) and TiO_2_ (50) NPs showed the same zones of inhibition against *E. coli* ATCC 25922, equal to 9 mm; the rest of the NPs gave no activity. Against *S. aureus* ATCC 25923, TiO_2_ (100) NPs were the only NP that showed a zone of inhibition equal to 10 mm. Gentamicin showed greater antibacterial activity than TiO_2_ NP (positive control) (Fig. S1 and S2). According to the previous results, TiO_2_ (100) NPs were the chosen NP for coating. As for Ag, TiO_2_ (100), AgTiO_2_ (0.5:1), AgTiO_2_ (1:1), AgTiO_2_ (1.5:1), AgTiO_2_ (2:1) NPs, they demonstrated greater sensitivity against Gram-positive bacteria (MRSA) than against Gram-negative bacteria, and a greater activity against *S.* Typhi serotype than *K. pneumoniae* was exhibited. Also, these NPs showed antibacterial activity against MDR bacteria stronger than that of ciprofloxacin (positive control). The AgTiO_2_ (2:1) NPs appeared to have the most superior activity against all bacteria, with zones of inhibition equal to 27 mm against MRSA, 15 mm against *K. pneumoniae*, and 17 mm against *S.* Typhi. So, the antibacterial activity increased with the Ag content increase in the samples (Fig. S3 and S4).

### Minimum inhibitory concentration (MIC) and minimum bactericidal concentration (MBC)

**Fig. 7:**
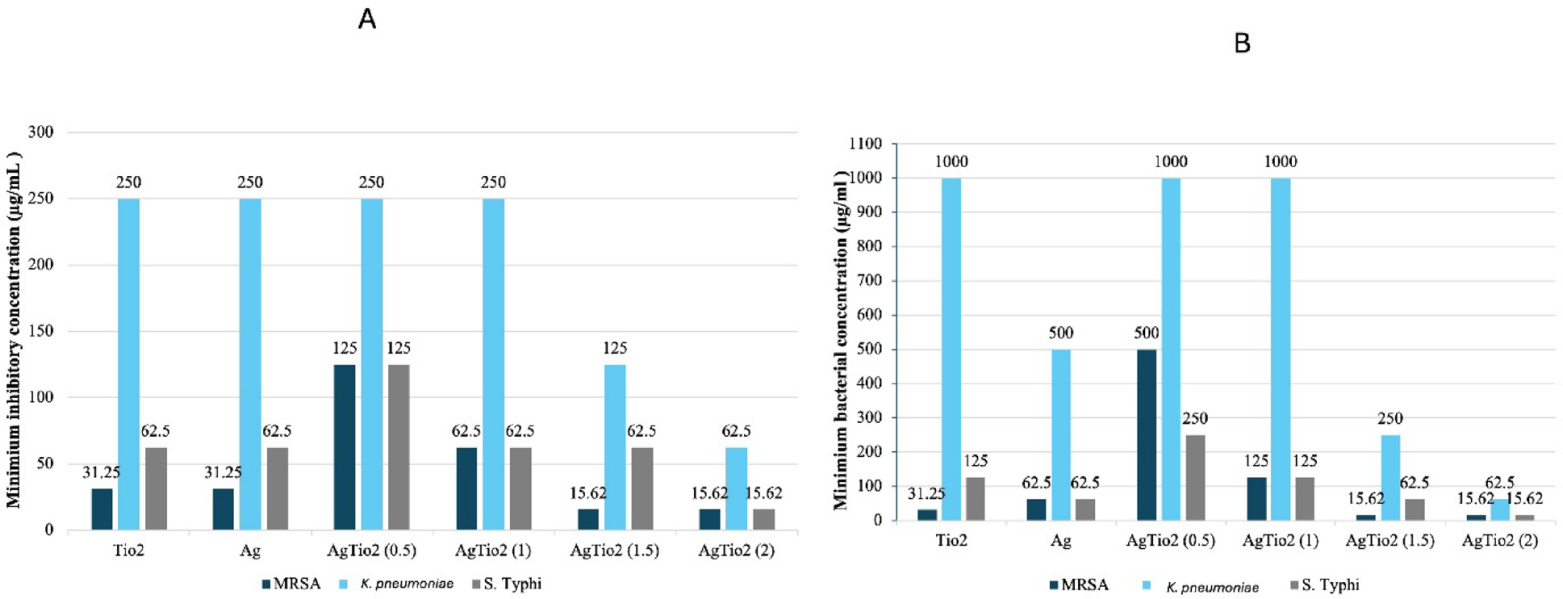
**A** Minimum Inhibitory Concentration (MIC) of NPs against multidrug-resistant strains, MRSA, *K. pneumoniae,* and *S.* Typhi serotype. **B** Minimum Bactericidal Concentration (MBC) of NPs against multidrug-resistant strains, MRSA*, K. pneumoniae,* and *S.* Typhi serotype

The results showed that Ag, TiO_2_ (100), AgTiO_2_ (0.5:1, AgTiO_2_ (1:1), AgTiO_2_ (1.5:1), AgTiO_2_ (2:1) NPs exhibit activity against MDR strains; MRSA*, K. pneumoniae,* and *S.* Typhi serotype, with MIC values ranging from 15.62 to 250 µg/mL shown in Fig. 7A and MBC from 15.62 to 1000 µg/mL in Fig. 7B. AgTiO_2_ (1.5:1) and AgTiO_2_ (2:1) NPs showed the same MIC and MBC against MRSA (15.62 µg/mL) which was the most powerful in comparison with the other tested NPs. AgTiO_2_ (2:1) NP had the lowest MIC and MBC against *K. pneumoniae* (62.5 µg/mL) and *S.* Typhi serotype (15.62 µg/mL). MBC and MIC were the same for AgTiO_2_ (2:1) against all bacteria, while the rest of the tested samples showed higher MBC values than MIC.

### Anti-biofilm activity of NPs

The biofilm inhibitory effect of Ag, TiO_2_ (100), AgTiO_2_ (0.5:1), AgTiO_2_ (1:1), AgTiO_2_ (1.5:1), AgTiO_2_ (2:1) NPs using different concentrations of NPs (75%, 50%, and 25% of MBC) against MDR strains; MRSA*, K. pneumoniae,* and *S.* Typhi serotype, is depicted in (Tables S2–S4). The above-mentioned NPs showed superior anti-biofilm activity against MRSA *compared* to *S.* Typhi serotype and *K. pneumoniae*. High biofilm inhibition percent appeared by all NPs (79.5–96.6%). The AgTiO_2_ (2:1) NP exhibited the strongest anti-biofilm activity against all bacterial isolates. It is observed that the greater the concentration and the higher the silver content in the sample, the greater the anti-biofilm activity.

### Cytotoxic effect of NPs

**Fig. 8:**
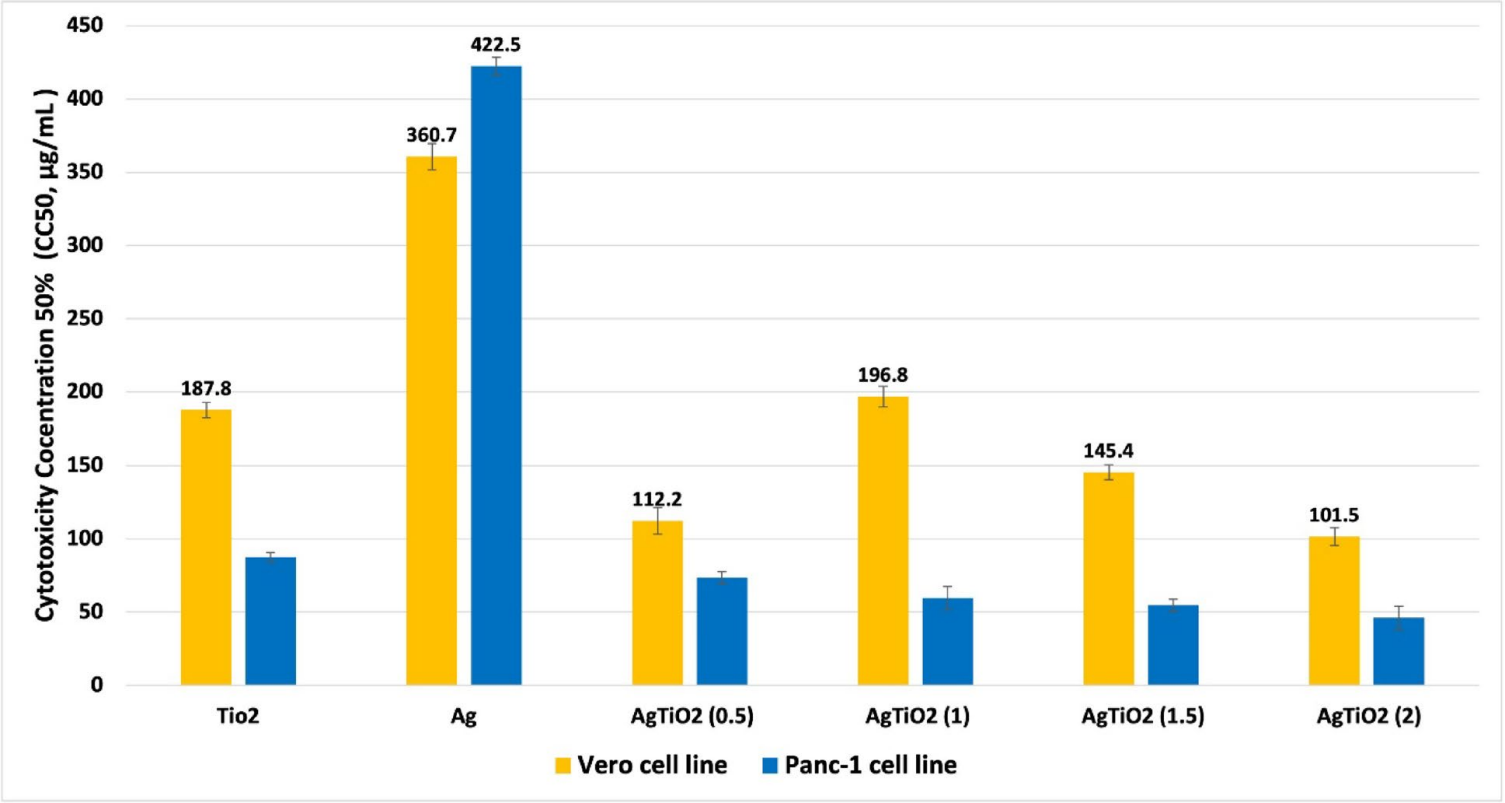
The cytotoxic concentration 50% (CC50, µg/mL) of nanoparticles (NPs) on *Vero* and *Panc-1* cell Line viability

The *Vero* (African Green Monkey Cercopithecus Aethiops Kidney Epithelial normal cell) (ATCC, Rockville, MD) and *Panc-1* (human pancreatic cancer cell) cell lines were provided by VACSERA, Giza, Egypt. With the use of 1 × 10^5^ cells/mL (100 µL/well), various concentrations of NPs (31.25–1000 μg/mL) were tested to see whether the synthesized NPs had any cytotoxic effect. Cytotoxicity is defined as a reduction in cell viability by greater than 30 percent compared with an untreated control. That means all synthesized NPs are cytotoxic, as all NPs decreased cell viability by more than 30% at different concentrations compared to the untreated control, which showed 100% viability. NPs showed less cytotoxicity against *Vero* cells in comparison to *Panc-1* cells, so NPs were observed with selective cytotoxicity (Fig. 8). The *Vero* and *Panc-1* cell lines were reduced in a dose-dependent manner, as shown in Tables S5 and S6. The AgTiO_2_ (2:1) NPs revealed the highest cytotoxic effect against *Vero* and *Panc-1* cell lines. As for Pure NPs, TiO_2_ NPs revealed a cytotoxic effect more than Ag NPs against both cell lines. (Figs. S5-S16).

## Discussion

The rise of multidrug-resistant (MDR) bacteria, such as MRSA, *K. pneumoniae*, and *S.* Typhi, due to misuse and abuse of antibacterial agents, poses a serious global health threat (Hamed et al. 2018). The development of alternative antibacterial agents, such as NPs, has gained attention due to their unique physicochemical properties and antibacterial activity (Banoub et al. 2021). Previous studies reported Ag NPs synthesis from *A. vera* as an antibacterial agent (Rajkumari et al. 2019; Ahmad et al. 2022) and reported TiO₂ NPs synthesis from *A. vera* with antibiofilm activity (Rajkumari et al. 2019). In this study, green-synthesized AgTiO_2_ NPs were introduced as a promising candidate for notable antibacterial, antibiofilm, and cytotoxic activity. Green synthesis offers an eco-friendly, cost-effective, and biocompatible approach to NPs production (Ibrahim et al. 2025). *A. vera* was selected as a reducing agent as it contains approximately 110 potentially active constituents from six different classes (Kahramanoğlu et al. 2019), which contribute to both NPs formation from metal ions and enhanced antibacterial activity, in line with earlier findings (Rajkumari et al. 2019; Hariharan et al. 2020).

In this study, synthesized NPs showed irregular spheres and polygonal shapes. Hariharan et al. synthesized AgTiO_2_ NPs using *A. vera,* introducing spherical Ag NPs and rod-shaped TiO_2_ NPs (Hariharan et al. 2020). Rathi et al. synthesized spherical AgTiO_2_ NPs using *Caesalpinia pulcherrima* flower extract as a reducing agent (Rathi et al. 2023). These different results are attributed to using different methods and parameters in NPs synthesis (Rathi et al. 2023). The size and shape of synthesized NPs are influenced by different experimental parameters as reactant concentrations, pH, temperature, reaction time, reducing agent, reactants used, and other parameters (Radulescu et al. 2023). The smaller the size of NPs, the larger the surface area exposed, enhancing their ability to penetrate bacterial cells and disrupt internal structures more effectively (Babu and Kim 2023). Different shapes interact with biological systems differently, as the NPs with sharp edges can cause more damage to cells. SEM and TEM analysis showed aggregated NPs of a size range of 20–70 nm for AgTiO_2_ NPs, 49nm and 50–70 nm for pure Ag NPs and TiO_2_ NPs, respectively. The aggregation may be attributed to the use of plant extract as a reducing agent (Rajkumari et al. 2019; Arshad et al. 2022). Some AgTiO_2_ NPs showed a grain size approximately less than that of pure forms. This may be due to the aggregation of particles in the pure NPs, then when exposed to synthesis again for composite formation (AgNO_3_ was added to pre-synthesized TiO_2_ NPs), aggregation decreased, decreasing grain size.

According to the well diffusion agar results, green-synthesized NPs had antibacterial activity against MDR bacteria stronger than that of the ciprofloxacin positive control. The current results agree with previous studies (Alsamman et al. 2023; Roopan et al. 2013). The results show that MRSA was the most susceptible clinical strain to NPs, followed by *S.* Typhi and then by *K. pneumoniae*. The difference in susceptibility between *S.* Typhi and *K. pneumoniae* may be due to the different mechanisms of resistance and the distinct cell wall compositions of these bacteria, even though both are Gram-negative. As for the advanced resistance of Gram-negative bacteria in comparison to Gram-positive bacteria, it may be due to the absence of an outer membrane in Gram-positive. Any change to the outer membrane as porin down-regulation or alteration of hydrophobicity, any change can cause bacterial resistance (Rodrigues et al. 2024). The combined AgTiO_2_ NPs showed enhanced antibacterial activity over the individual NPs. This synergistic effect can be attributed to the combined antibacterial properties of TiO_2_ and Ag NPs (Alsamman et al. 2023). Among the AgTiO_2_ NPs, AgTiO_2_ (2:1) NPs exhibited the most significant zone of inhibition against the tested bacteria. In line with well diffusion agar results, the MIC, MBC, and Antibiofilm assays revealed that MRSA showed the greatest susceptibility to NPs, followed by *S.* Typhi serotype, then *K. pneumoniae* (Hidayat et al. 2024). The AgTiO₂ (2:1) NPs had the most powerful MIC, MBC, and biofilm inhibition. Showing equal MIC and MBC is in line with Parvekar et al. study. Parvekar et al. demonstrated that MIC and MBC of Ag NPs against *S. aureus* are the same and equal to 0.625 mg/mL (Parvekar et al. 2020). Ratshiedana et al. introduced 0.2% Ag-TiO_2_ NPs of high activity against Gram-positive bacteria compared to the Gram-negative bacteria. The MIC values observed against Gram-positive *B. subtilis* and *S. aureus* were 1250 and 620 µg/mL*,* respectively. On the other hand, gram-negative *S. typhimurium* and *E. coli* showed MIC values greater than 2500 µg/mL (Ratshiedana et al. 2024).

The proposed antibacterial mechanism of NPs involves the attachment of TiO_2_ NPs and Ag⁺ ions to the bacterial cell wall, leading to the disruption of key metabolic processes. The adhesion of Ag⁺ ions to the cell surface compromises membrane integrity, allowing the ions to penetrate the cell and subsequently inactivate and/or modify bacterial enzymes and damage DNA or generate reactive oxygen species (ROS) within microbial cells (Nawaz et al. 2024; Babu and Kim 2023). TiO_2_ NPs exert their antibacterial effect through the generation of reactive oxygen species (ROS) within microbial cells, leading to lipid peroxidation of extracellular polymeric substances (EPS) and oxidation of intracellular enzymes. These processes disrupt cellular respiration and ultimately induce apoptosis (Bereanu et al. 2024). But the exact mechanisms are still unknown (Serov et al. 2024). Moreover, the phytochemicals from *A. vera* not only aid in NPs synthesis but also enhance antibacterial efficacy by damaging bacterial cell walls, inactivating enzymes, interacting with bacterial proteins and lipids, and disrupting of replication of bacterial DNA (Subramani et al. 2018).

MTT assay showed that all NPs were cytotoxic with relatively selective cytotoxicity against cancerous *Panc-1* cells, more than normal *Vero* cells. Ag NP was the only sample that showed greater cytotoxicity against *Vero* cells. As for Pure NPs, TiO_2_ NP revealed cytotoxic effect more than Ag NP against both cell lines. Although coated NPs revealed higher cytotoxicity as the silver content increased in the samples. These results are due to different shapes and sizes of NPs, which affect their activity (Thu et al. 2023). The suggested cytotoxic effect of NPs involves apoptosis, angiogenesis suppression, damage to the membrane or mitochondria or DNA, and interference in protein synthesis (Alshameri and Owais 2022). NPs appeared to be promising cytotoxic drugs. But further modification to the physical and chemical properties of NPs is necessary to decrease their side effects.

After testing NPs microbiologically, AgTiO_2_ (2:1) NPs seemed to be the best sample, so further characterization was performed on it. TEM images of AgTiO_2_ (2:1) NPs confirmed shape, average grain size, and successful coating of Ag NPs on TiO_2_ NPs (Alsamman et al. 2023; Nagaraj et al. 2023). Selected area electron diffraction (SAED) patterns displayed bright concentric rings, confirming the polycrystalline nature of NPs. Energy dispersion X-ray spectroscopy (EDX) confirmed the presence of chemical elements 57.31% Ag, 29.47% Ti, 10.01% O, 3.02% C, and 0.01% N with right percentages (Hariharan et al. 2020). Carbon is from carbon-coated grade used in capturing sample by Jeol (JEM-2100 PLUS, Akishima, Tokyo, Japan), and part of the carbon is from *A. vera,* while nitrogen is from *A. vera* used (Hossain et al. 2020), so successful coating took place with approximately the right percentage Ag: TiO_2_ (2:1).

Our findings are in line with prior research highlighting the enhanced efficacy of nanocomposites over monometallic NPs. However, the use of *A. vera* for green synthesis in this study provided the additional benefit of reduced toxicity and improved biocompatibility, which are critical for potential clinical applications. The successful coating of TiO_2_ NPs with varying ratios of Ag NPs allowed us to optimize antibacterial performance, with AgTiO_2_ (2:1) NPs proving most effective across all assays. In summary, our study successfully synthesized Ag, TiO_2_, and AgTiO_2_ NPs via a green, cost-effective method using *A. vera* extract. The antibacterial, antibiofilm, and cytotoxic effects were investigated, revealing that all NPs were of good antibacterial and antibiofilm activity, while AgTiO_2_ (2:1) NP activity was the most superior. It is concluded that green-synthesized Ag and TiO_2_ NPs have a synergistic effect, especially at high Ag concentrations. NPs maintained low cytotoxicity toward normal *Vero* cells compared to cancerous *Panc*-1 cells. On the other hand, all materials used in synthesis were eco-friendly, common, and low-cost. The synergistic antibacterial and antibiofilm properties, combined with acceptable cytotoxicity and cost-effective synthesis, indicate that these green-synthesized NPs are a promising alternative to therapeutic agents for managing MDR bacterial infections, especially in healthcare settings where biofilm-associated infections pose major challenges. Despite the promising results, our study has certain limitations. Specifically, the mechanisms of action of Ag and TiO_2_ NPs against bacteria and cytotoxicity require deeper investigation. Further research, including preclinical and clinical evalution, are required to clarify the underlying mechanisms, including more bacterial strains, and assess the safety and long-term effectiveness of AgTiO₂ NPs in appropriate biological models. Enhancing the scalability and reproducibility of the biosynthesis process will also be crucial for advancing the practical and clinical applications of these NPs across various fields.

## Supplementary Information

Below is the link to the electronic supplementary material.

Supplementary Material 1

## Data Availability

All data generated or analyzed during this study are included in this published article and supplementary file.
