# Supplementary material for: Antibacterial, antibiofilm and cytotoxic properties of Aloe vera-synthesized silver titanium nanoparticles
Source: AMB Express. 2025 Oct 27;15:157. doi: 10.1186/s13568-025-01965-8 (PMC12559554; doi:10.1186/s13568-025-01965-8)
Supplement: Supplementary file 1 — Supplementary Material 1 [file 13568_2025_1965_MOESM1_ESM.docx]

**Table S1.** Crystallite domain size, dislocation density and lattice strain results of the prepared NPs.

| **Samples** | **Mean values crystallite domain size (** **D, (nm)** | **Mean values of dislocation density (δ, (nm)** | **Mean values of lattice strain(ε)** |
| --- | --- | --- | --- |
| TiO_2_  (1) | 30.26 | 0.002266 | 0.001515 |
| TiO_2_ (25) | 53.31576 | 0.000593 | 0.000764 |
| TiO_2_ (50) | 31.96782 | 0.002958 | 0.001709 |
| TiO_2_ (100) | 21.45325 | 0.003912 | 0.001987 |
| Ag | 52.70601 | 0.00059 | 0.000795 |
| AgTiO_2_ (0.5:1) | 36.28964 | 0.003191 | 0.001654 |
| AgTiO_2_ (1:1) | 53.3583 | 0.001356 | 0.001 |
| AgTiO_2_ (1.5:1) | 56.15144 | 0.002097 | 0.001284 |
| AgTiO_2_ (2:1) | 53.41652 | 0.002367 | 0.001264 |

**Table S2.** Biofilm inhibition percentage (%) of NPs concentrations (µg/mL), equivalent to 25% of their MBC, used against MDR strains; MRSA*, K. pneumoniae,* and *S. Typhi* serotype.

| **Tested NPs** | | **MRSA** CCASU-11 | ***K. pneumoniae*** CCASU-24 | ***S.* Typhi** CCASU-31 |
| --- | --- | --- | --- | --- |
| TiO_2_ (100) | concentration (µg/mL) | 7.81 | 250 | 31.25 |
|  | inhibition percentage (%) | 83.37 | 87.98 | 90.01 |
| Ag | concentration (µg/mL) | 15.6 | 125 | 15.6 |
|  | inhibition percentage (%) | 82.68 | 88.77 | 87.04 |
| AgTiO_2_ (0.5) | concentration (µg/mL) | 125 | 250 | 62.5 |
|  | inhibition percentage (%) | 79.91 | 84.62 | 79.52 |
| AgTiO_2_ (1) | concentration (µg/mL) | 31.25 | 250 | 31.25 |
|  | inhibition percentage (%) | 81.15 | 89.02 | 82.43 |
| AgTiO_2_ (1.5) | concentration (µg/mL) | 3.9 | 62.5 | 15.6 |
|  | inhibition percentage (%) | 83.13 | 90.26 | 84.06 |
| AgTiO_2_ (2) | concentration (µg/mL) | 3.9 | 15.6 | 3.9 |
|  | inhibition percentage (%) | 87.07 | 92.81 | 85.98 |

**Table S3.** Biofilm inhibition percentage (%) of NPs concentrations (µg/mL), equivalent to 50% of their MBC, used against MDR MRSA*, K. pneumoniae,* and *S. Typhi* serotype.

| **Tested NPs** | | **MRSA** CCASU-11 | ***K. pneumoniae*** CCASU-24 | ***S.* Typhi** CCASU-31 |
| --- | --- | --- | --- | --- |
| TiO_2_ (100) | concentration (µg/mL) | 15.63 | 500 | 62.5 |
|  | inhibition percentage (%) | 89.84 | 92.4 | 92.81 |
| Ag | concentration (µg/mL) | 31.25 | 250 | 31.25 |
|  | inhibition percentage (%) | 88.08 | 92.46 | 91.08 |
| AgTiO_2_ (0.5) | concentration (µg/mL) | 250 | 500 | 125 |
|  | inhibition percentage (%) | 85.72 | 87.84 | 83.26 |
| AgTiO_2_ (1) | concentration (µg/mL) | 62.5 | 500 | 62.5 |
|  | inhibition percentage (%) | 86.19 | 91.46 | 86.72 |
| AgTiO_2_ (1.5) | concentration (µg/mL) | 7.81 | 125 | 31.25 |
|  | inhibition percentage (%) | 87.45 | 92.45 | 88.74 |
| AgTiO_2_ (2) | concentration (µg/mL) | 7.81 | 31.25 | 7.81 |
|  | inhibition percentage (%) | 90.39 | 94.63 | 91.07 |

**Table S4.** Biofilm inhibition percentage (%) of NPs concentrations (µg/mL), equivalent to 75% of their MBC, used against MDR MRSA*, K. pneumoniae,* and *S. Typhi* serotype.

| **Tested NPs** | | **MRSA** CCASU-11 | ***K. pneumoniae*** CCASU-24 | ***S.* Typhi** CCASU-31 |
| --- | --- | --- | --- | --- |
| TiO_2_ (100) | concentration (µg/mL) | 23.44 | 750 | 93.75 |
|  | inhibition percentage (%) | 94.27 | 95.67 | 96.2 |
| Ag | concentration (µg/mL) | 46.88 | 375 | 46.88 |
|  | inhibition percentage (%) | 92.88 | 95.95 | 95.87 |
| AgTiO_2_ (0.5) | concentration (µg/mL) | 375 | 750 | 187.5 |
|  | inhibition percentage (%) | 90.74 | 92 | 88.53 |
| AgTiO_2_ (1) | concentration (µg/mL) | 93.75 | 750 | 93.75 |
|  | inhibition percentage (%) | 91.4 | 94.18 | 90.38 |
| AgTiO_2_ (1.5) | concentration (µg/mL) | 11.7 | 187.5 | 46.88 |
|  | inhibition percentage (%) | 92.21 | 94.95 | 92.51 |
| AgTiO_2_ (2) | concentration (µg/mL) | 11.7 | 46.875 | 11.7 |
|  | inhibition percentage (%) | 94.96 | 96.65 | 94.13 |

**Table. S5.** Effect of Ag, TiO_2_ (100), AgTiO_2_ (0.5:1), AgTiO_2_ (1:1), AgTiO_2_ (1.5:1), AgTiO_2_ (2:1) NPs on *Vero* cell viability at different concentrations.

| **Tested NPs** | **concentration (µg/mL)** | **Viability %** | **CC50%(µg/mL)** |
| --- | --- | --- | --- |
| TiO_2_ (100) | 1000 | 4 | 187.8 |
|  | 500 | 4.5 |  |
|  | 250 | 26 |  |
|  | 125 | 75 |  |
|  | 62.5 | 100 |  |
|  | 31.25 | 100 |  |
|  |  |  |  |
| Ag | 1000 | 7.5 | 360.7 |
|  | 500 | 30.16 |  |
|  | 250 | 66 |  |
|  | 125 | 99.9 |  |
|  | 62.5 | 100 |  |
|  | 31.25 | 100 |  |
|  |  |  |  |
| AgTiO_2_ (0.5:1) | 1000 | 3.16 | 112.5 |
|  | 500 | 2.98 |  |
|  | 250 | 2.98 |  |
|  | 125 | 45.54 |  |
|  | 62.5 | 75.39 |  |
|  | 31.25 | 98.55 |  |
|  |  |  |  |
| AgTiO_2_ (1:1) | 1000 | 2.85 | 196.8 |
|  | 500 | 5 |  |
|  | 250 | 30.5 |  |
|  | 125 | 71 |  |
|  | 62.5 | 95.9 |  |
|  | 31.25 | 99.8 |  |
|  |  |  |  |
| AgTiO_2_ (1.5:1) | 1000 | 2.6 | 145.4 |
|  | 500 | 3 |  |
|  | 250 | 27.3 |  |
|  | 125 | 53.3 |  |
|  | 62.5 | 56.2 |  |
|  | 31.25 | 80.9 |  |
|  |  |  |  |
| AgTiO_2_ (2:1) | 1000 | 3.1 | 101.5 |
|  | 500 | 2.75 |  |
|  | 250 | 7.3 |  |
|  | 125 | 46.87 |  |
|  | 62.5 | 54.6 |  |
|  | 31.25 | 65.8 |  |
|  |  |  |  |

**Table. S6.** Effect of Ag, TiO_2_ (100), AgTiO_2_ (0.5:1), AgTiO_2_ (1:1), AgTiO_2_ (1.5:1), AgTiO_2_ (2:1) NPs on *Panc-1* cell viability at different concentrations.

| **Tested NPs** | **concentration (µg/mL)** | **Cell viability %** | **CC50%(µg/mL)** |
| --- | --- | --- | --- |
| TiO_2_ (100) | 1000 | 2.25 | 87.5 |
|  | 500 | 2.34 |  |
|  | 250 | 2.38 |  |
|  | 125 | 18.9 |  |
|  | 62.5 | 68.88 |  |
|  | 31.25 | 99.65 |  |
|  |  |  |  |
| Ag | 1000 | 2.63 | 422.5 |
|  | 500 | 28.82 |  |
|  | 250 | 98.42 |  |
|  | 125 | 99.87 |  |
|  | 62.5 | 99.82 |  |
|  | 31.25 | 99.91 |  |
|  |  |  |  |
| AgTiO_2_ (0.5:1) | 1000 | 2.46 | 73.4 |
|  | 500 | 2.59 |  |
|  | 250 | 2.68 |  |
|  | 125 | 2.68 |  |
|  | 62.5 | 56.49 |  |
|  | 31.25 | 92.42 |  |
|  |  |  |  |
| AgTiO2 (1:1) | 1000 | 2.55 | 59.5 |
|  | 500 | 2.46 |  |
|  | 250 | 2.38 |  |
|  | 125 | 2.63 |  |
|  | 62.5 | 31.92 |  |
|  | 31.25 | 94.16 |  |
|  |  |  |  |
| AgTiO2 (1.5:1) | 1000 | 2.59 | 54.8 |
|  | 500 | 2.25 |  |
|  | 250 | 2.29 |  |
|  | 125 | 2.8 |  |
|  | 62.5 | 37.93 |  |
|  | 31.25 | 94.21 |  |
|  |  |  |  |
| AgTiO2 (2:1) | 1000 | 2.76 | 46.1 |
|  | 500 | 2.59 |  |
|  | 250 | 2.63 |  |
|  | 125 | 2.89 |  |
|  | 62.5 | 23.28 |  |
|  | 31.25 | 79.99 |  |


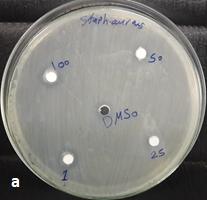

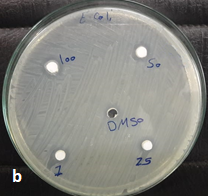


**Fig.S1**. Antibacterial activity of TiO_2_ (1), TiO_2_ (25), TiO_2_ (50), TiO_2_ (100) NPs against (a) *S. aureus* ATCC 25923 and (b) *E. coli* ATCC 25922 reference strains.

**Fig. S2.** Zones of inhibition of TiO_2_ (1), TiO_2_ (25), TiO_2_ (50), TiO_2_ (100) NPs against *E. coli* ATCC 25922 and *S. aureus* ATCC 25923 strains using gentamicin as positive control (control).


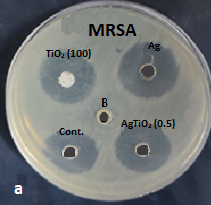

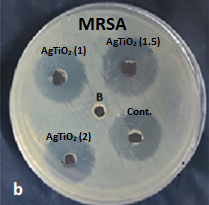


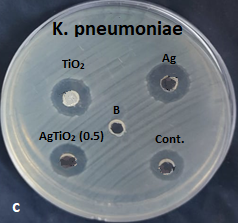

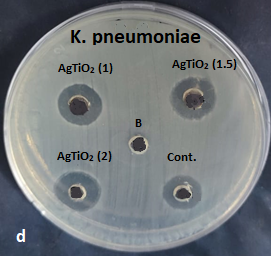


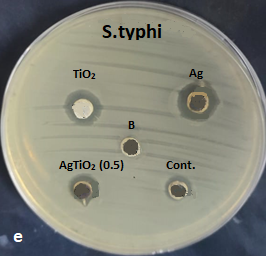

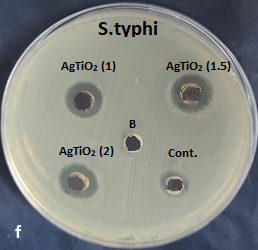


**Fig.S3.** Antibacterial activity of Ag, TiO_2_ (100), AgTiO_2_ (0.5:1), AgTiO_2_ (1:1), AgTiO_2_ (1.5:1), AgTiO_2_ (2:1) NPs against MDR (a-b) MRSA (c-d) *K. pneumoniae* (e-f) *S.* typhi serotype. Cont., ciprofloxacin, as positive control and B, distilled water, as negative control.


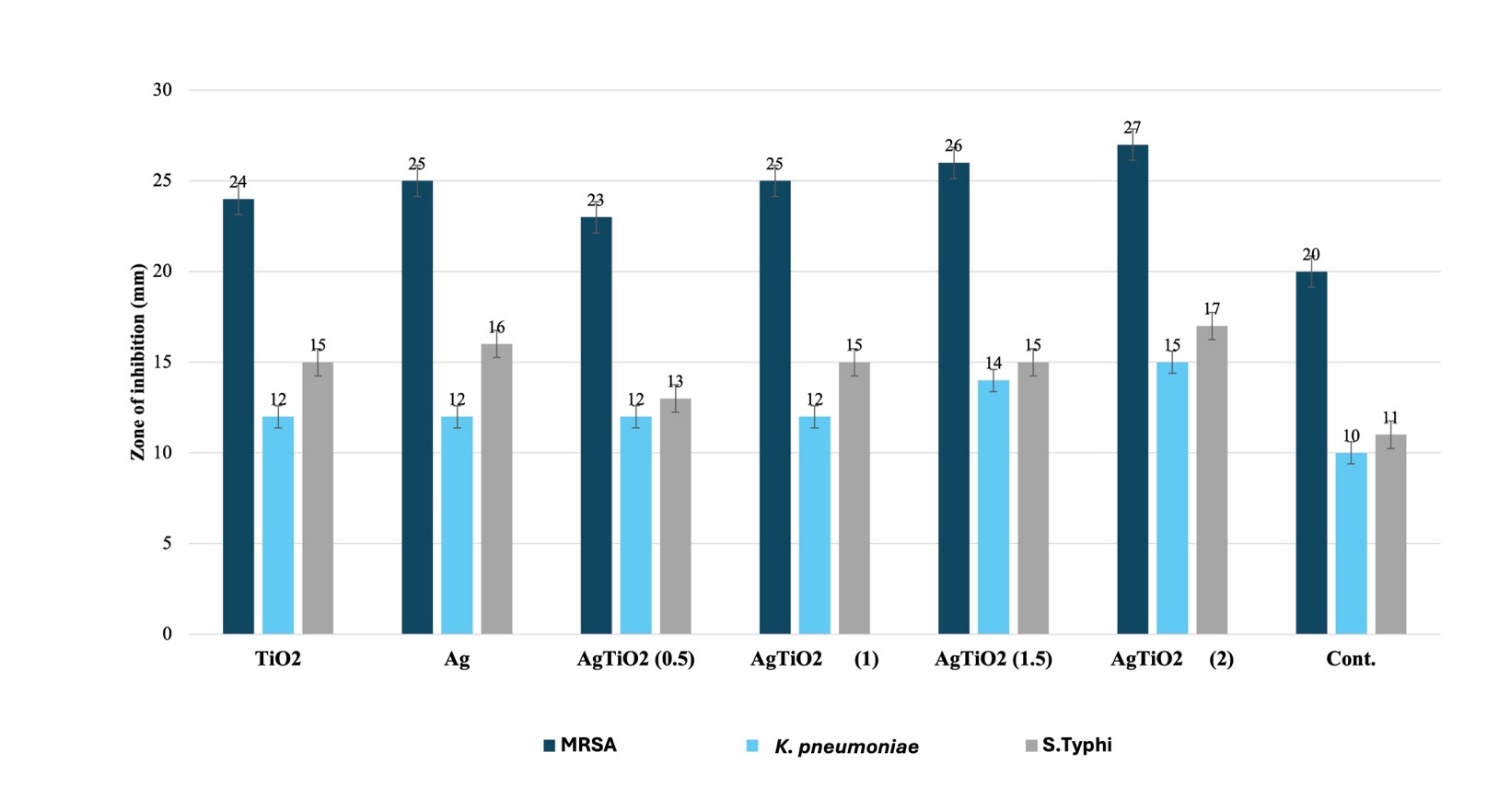


**Fig. S4.** Zones of inhibition of Ag, TiO_2_ (100), AgTiO_2_ (0.5:1), AgTiO_2_ (1:1), AgTiO_2_ (1.5:1), AgTiO_2_ (2:1) NPs against MDR MRSA CCASU-11*, K. pneumoniae* CCASU-24*,* and S. Typhi serotype CCASU-31 using ciprofloxacin as positive control (Cont.)**.**


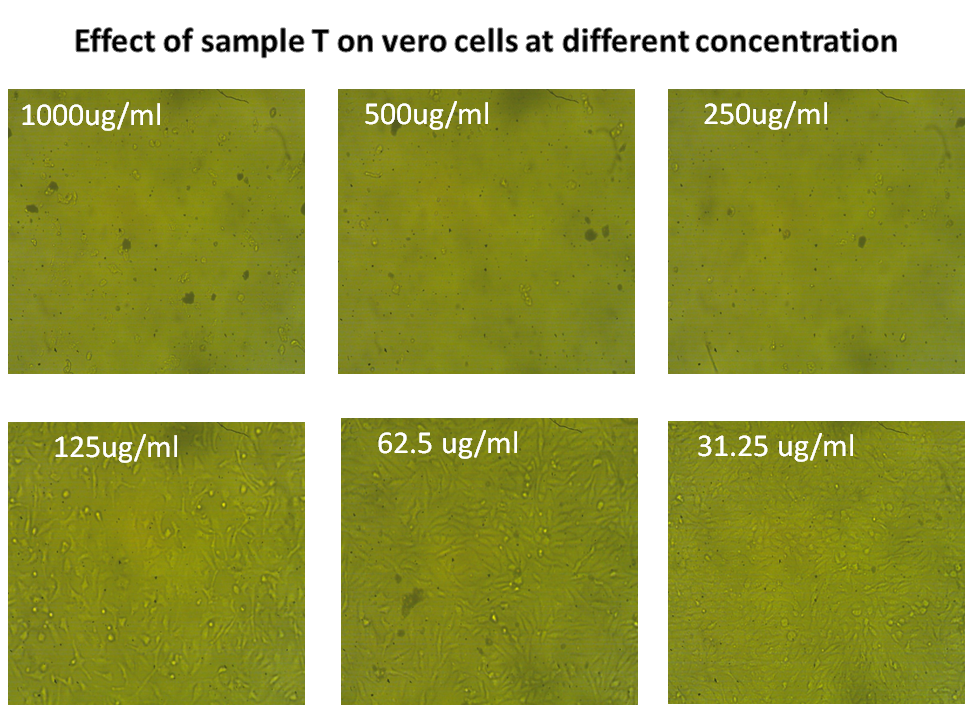


**Fig.S5.** Effect of TiO_2_ (100) NPs on *Vero* cell line at different concentrations.


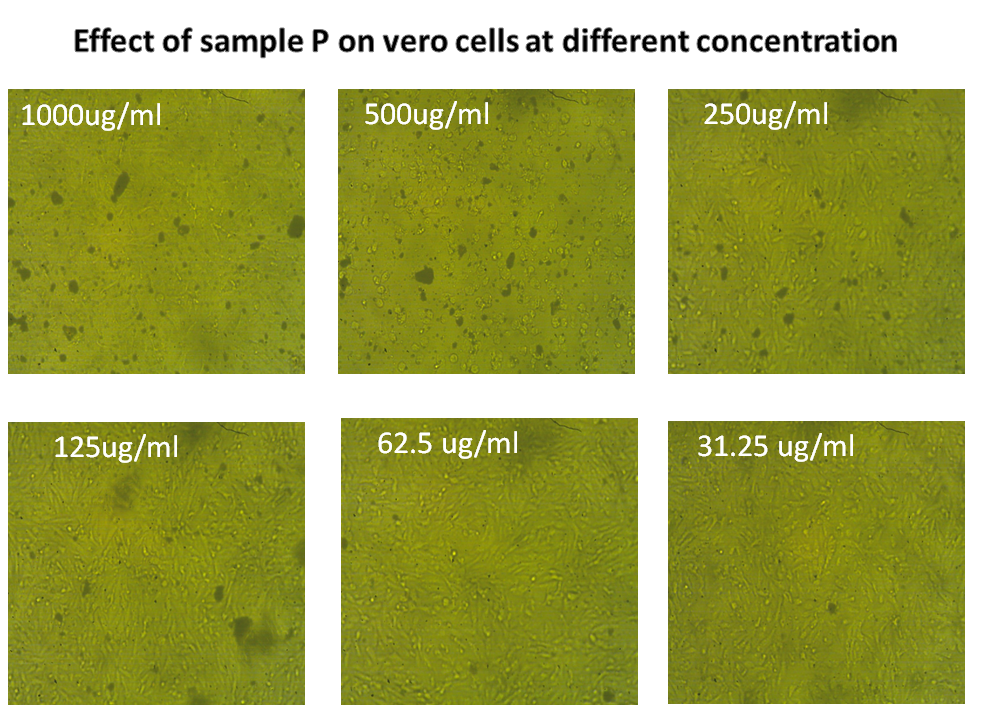


**Fig.S6.** Effect of Ag NPs on *Vero* cell line at different concentrations.


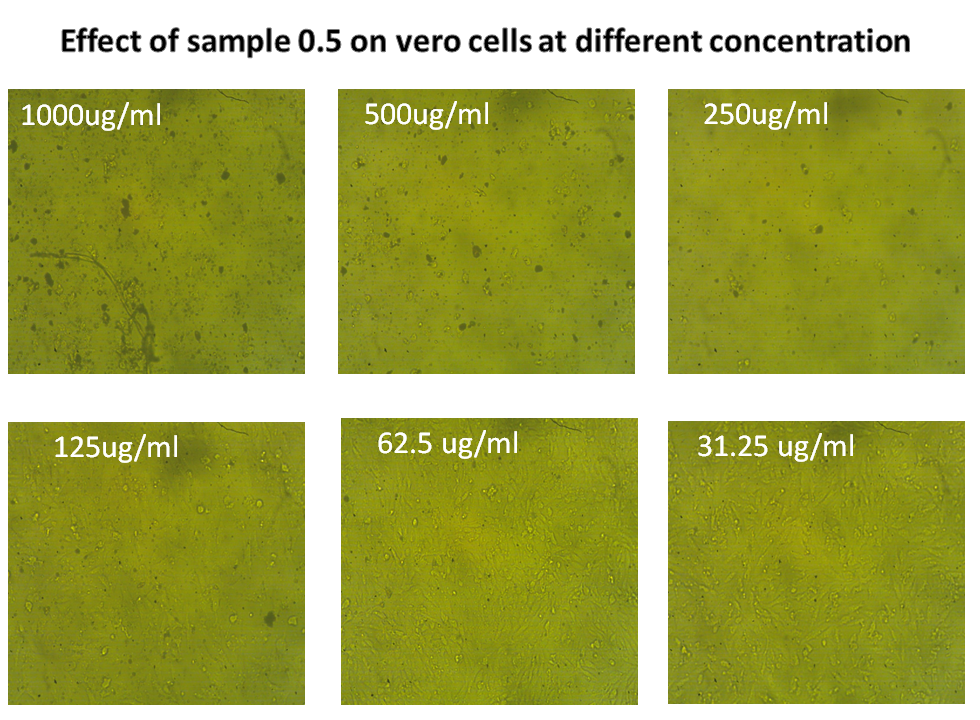


**Fig.S7.** Effect of AgTiO_2_ (0.5:1) NPs on *Vero* cell line at different concentrations.


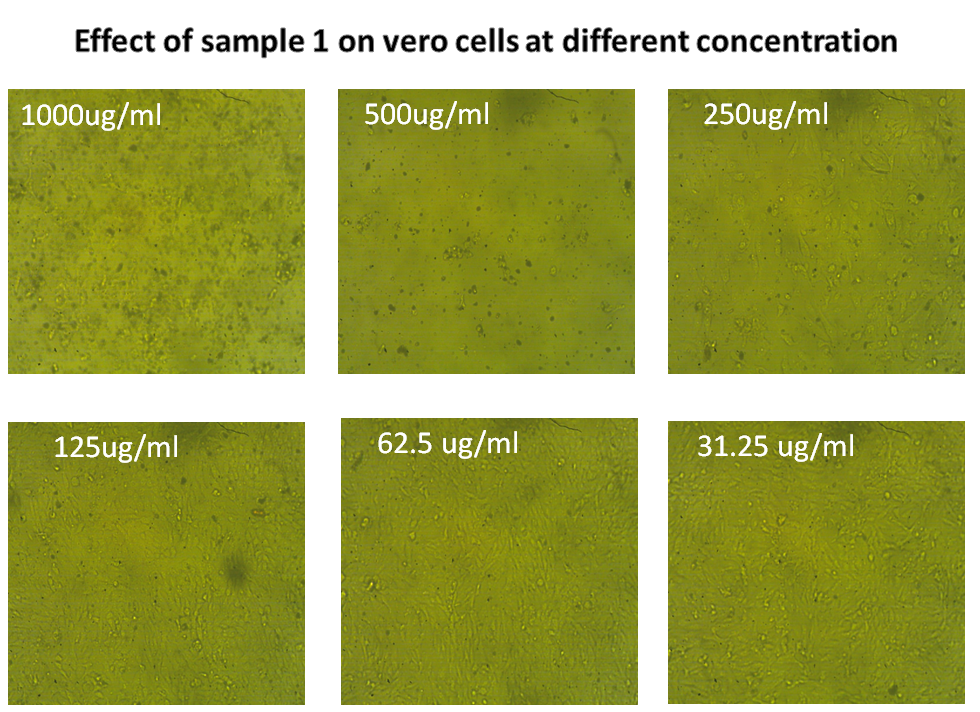


**Fig.S8.** Effect of AgTiO_2_ (1:1) NPs on *Vero* cell line at different concentrations.


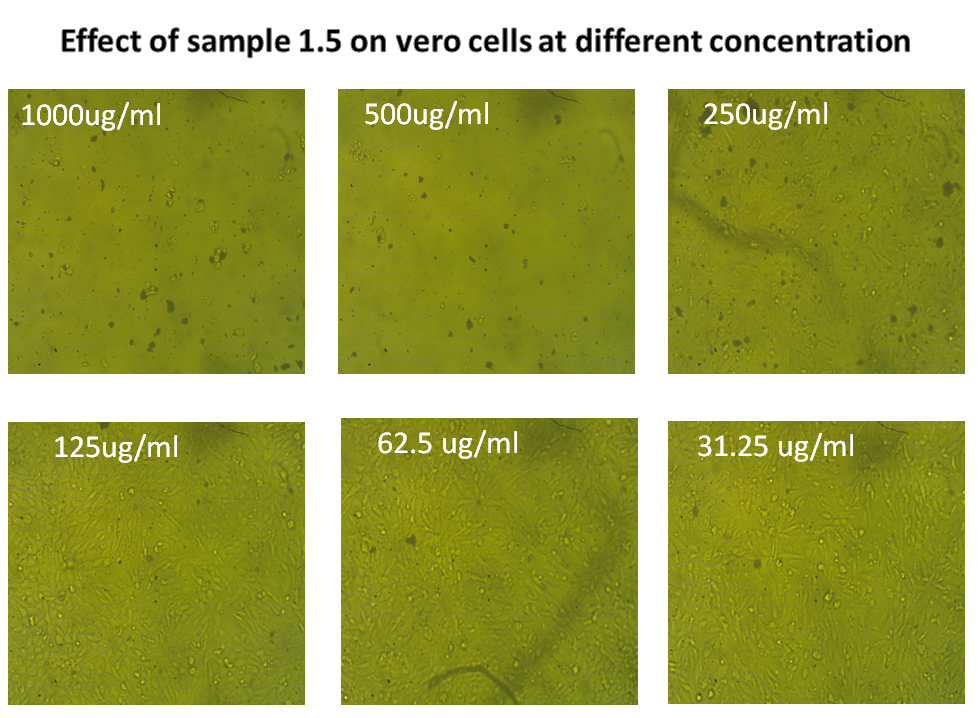


**Fig.S9.** Effect of AgTiO_2_ (1.5:1) NPs on *Vero* cell line at different concentrations.


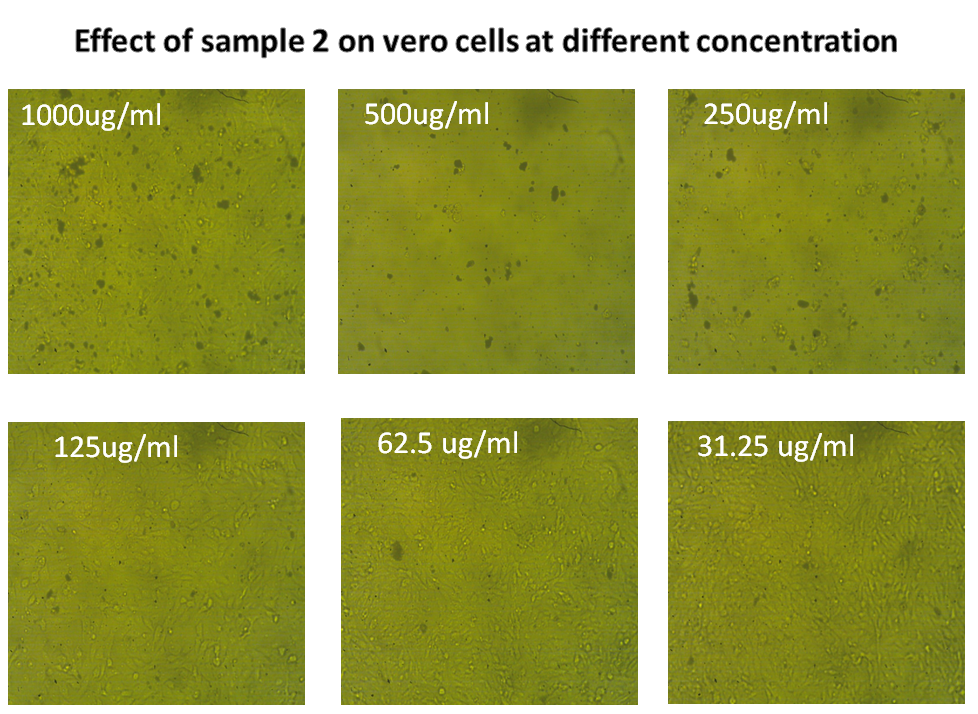


**Fig.S10.** Effect of AgTiO_2_ (2:1) NPs on *Vero* cell line at different concentrations.


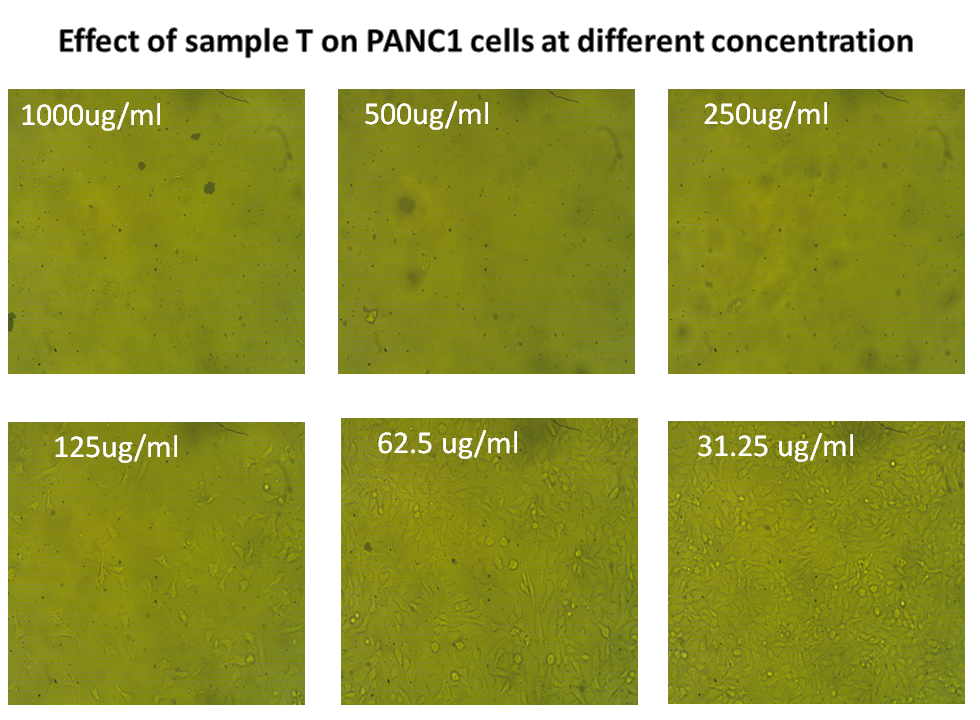


**Fig.S11.** Effect of TiO_2_ (100) NPs on *Panc-1* cell line at different concentrations.


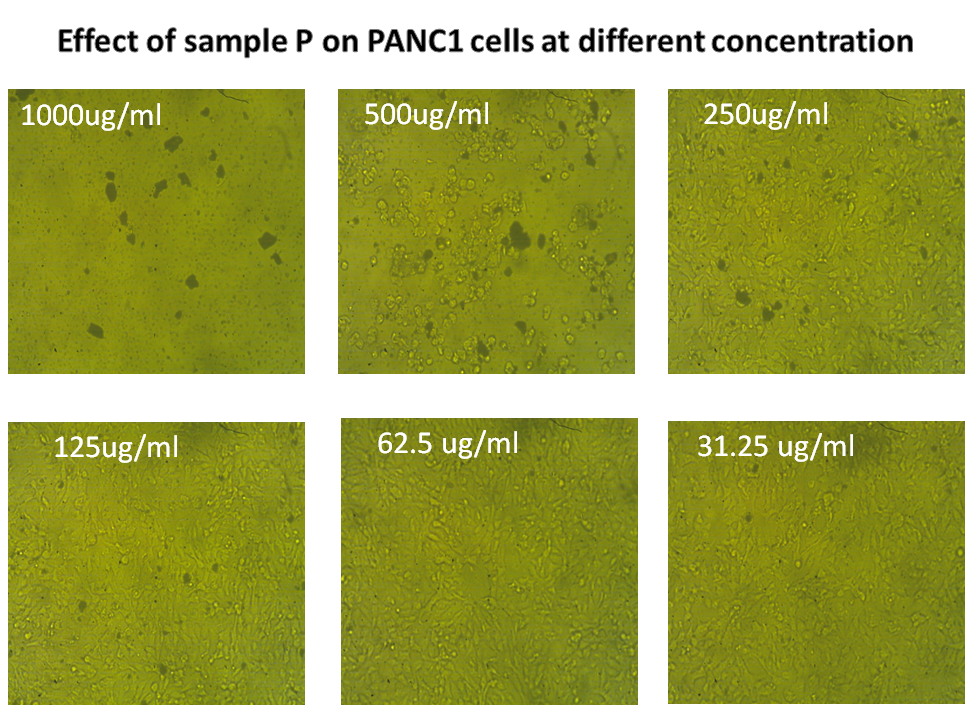


**Fig.S12.** Effect of Ag NPs on *Panc-1* cell line at different concentrations.


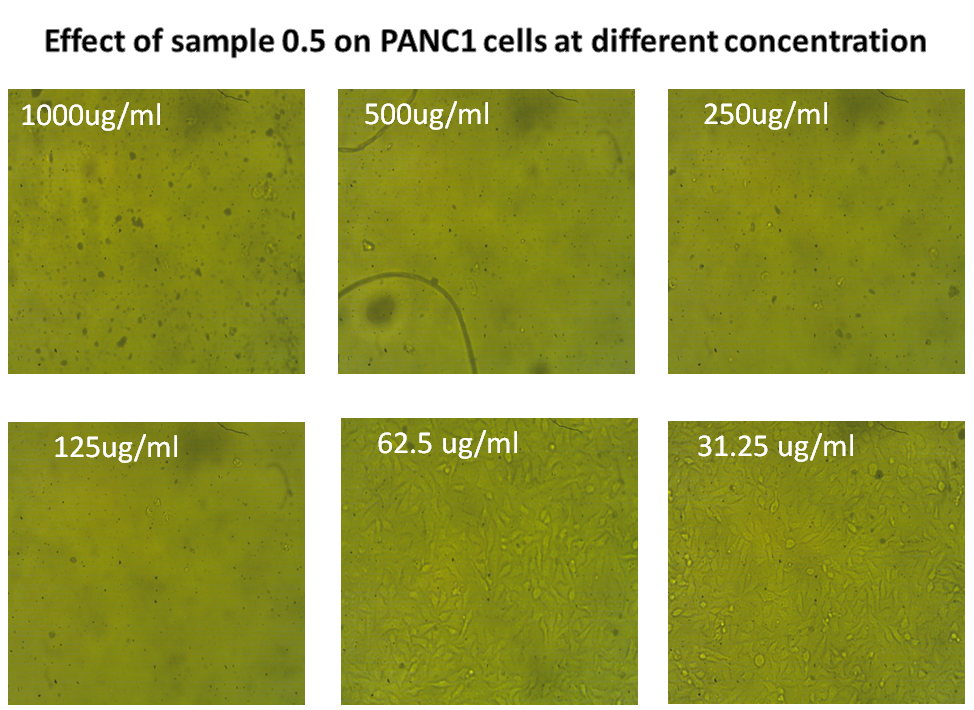


**Fig.S13.** Effect of AgTiO_2_ (0.5:1) NPs on *Panc-1* cell line at different concentrations.


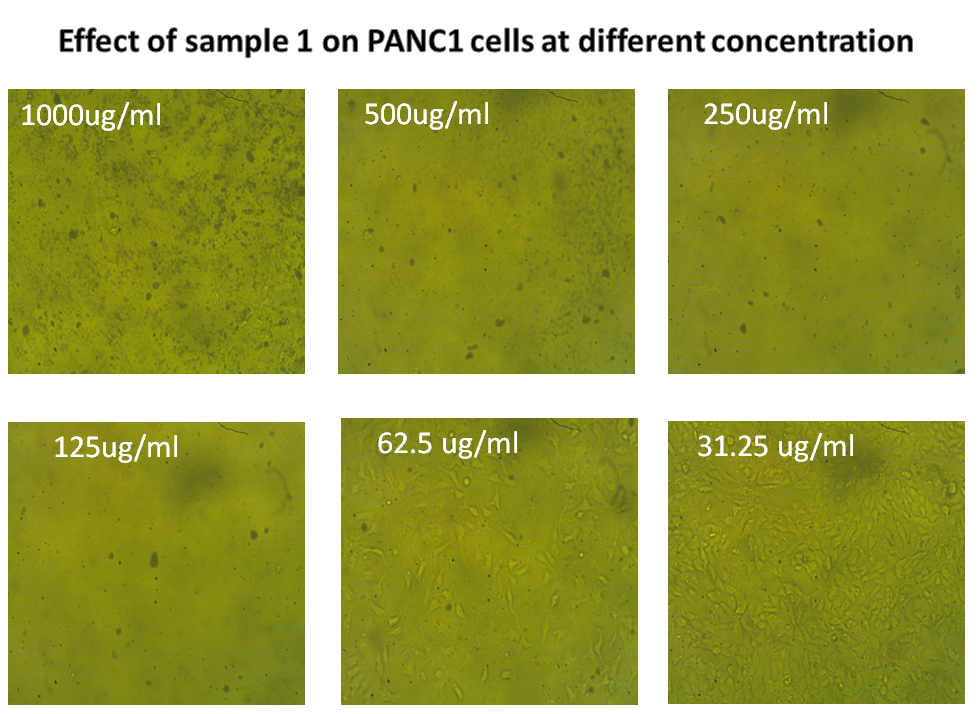


**Fig.S14.** Effect of AgTiO_2_ (1:1) NPs on *Panc-1* cell line at different concentrations.


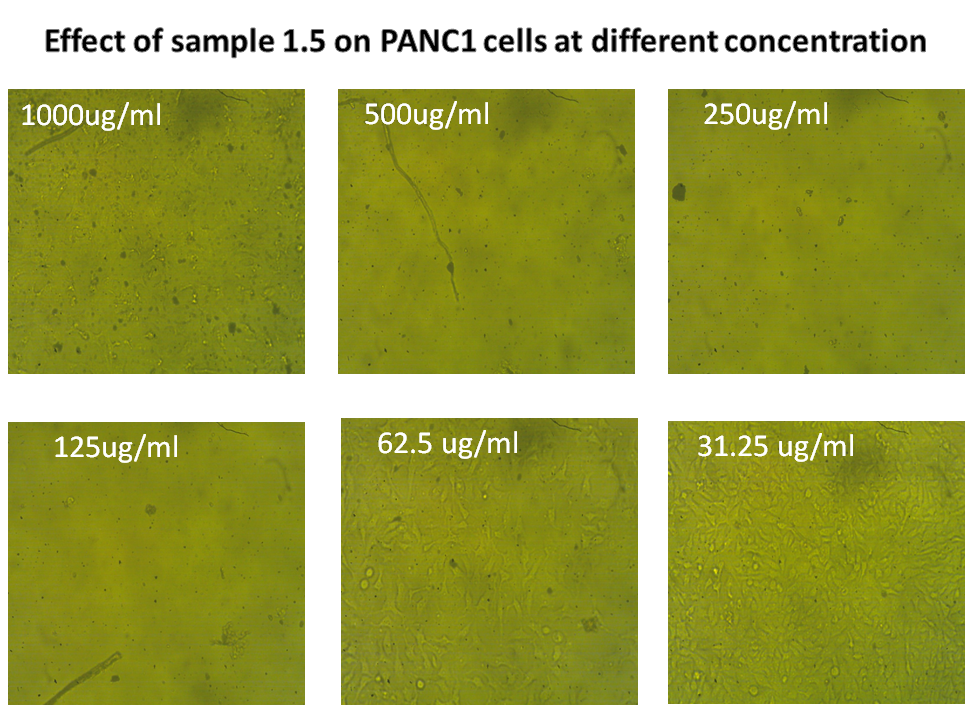


**Fig.S15.** Effect of AgTiO_2_ (1.5:1) NPs on *Panc-1* cell line at different concentrations.


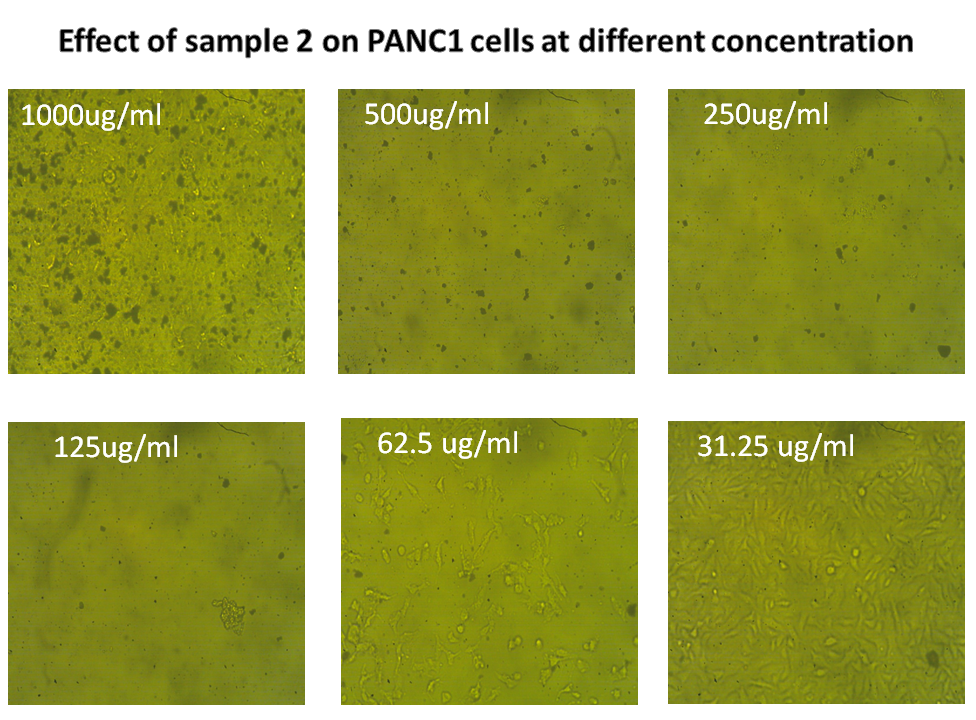


**Fig.S16.** Effect of AgTiO_2_ (2:1) NPs on *Panc-1* cell line at different concentrations.
